# HLA class I haplotype diversity is consistent with selection for frequent existing haplotypes

**DOI:** 10.1371/journal.pcbi.1005693

**Published:** 2017-08-28

**Authors:** Idan Alter, Loren Gragert, Stephanie Fingerson, Martin Maiers, Yoram Louzoun

**Affiliations:** 1 Department of Mathematics, Bar-Ilan University, Ramat Gan, Israel; 2 National Marrow Donor Program, Minneapolis, Minnesota, United States of America; 3 Department of Pathology and Laboratory Medicine, Tulane University School of Medicine, New Orleans, Louisiana, United States of America; University of Groningen, NETHERLANDS

## Abstract

The major histocompatibility complex (MHC) contains the most polymorphic genetic system in humans, the human leukocyte antigen (HLA) genes of the adaptive immune system. High allelic diversity in HLA is argued to be maintained by balancing selection, such as negative frequency-dependent selection or heterozygote advantage. Selective pressure against immune escape by pathogens can maintain appreciable frequencies of many different HLA alleles. The selection pressures operating on combinations of HLA alleles across loci, or haplotypes, have not been extensively evaluated since the high HLA polymorphism necessitates very large sample sizes, which have not been available until recently. We aimed to evaluate the effect of selection operating at the HLA haplotype level by analyzing HLA A~C~B~DRB1~DQB1 haplotype frequencies derived from over six million individuals genotyped by the National Marrow Donor Program registry. In contrast with alleles, HLA haplotype diversity patterns suggest purifying selection, as certain HLA allele combinations co-occur in high linkage disequilibrium. Linkage disequilibrium is positive (*D*_*ij*_'>0) among frequent haplotypes and negative (*D*_*ij*_'<0) among rare haplotypes. Fitting the haplotype frequency distribution to several population dynamics models, we found that the best fit was obtained when significant positive frequency-dependent selection (FDS) was incorporated. Finally, the Ewens-Watterson test of homozygosity showed excess homozygosity for 5-locus haplotypes within 23 US populations studied, with an average *Fnd* of 28.43. Haplotype diversity is most consistent with purifying selection for HLA Class I haplotypes (HLA-A, -B, -C), and was not inferred for HLA Class II haplotypes (-DRB1 and—DQB1). We discuss our empirical results in the context of evolutionary theory, exploring potential mechanisms of selection that maintain high linkage disequilibrium in MHC haplotype blocks.

## Author summary

The adaptive immune system presents antigens derived from pathogenic and normal self proteins on the cell surface using human leukocyte antigen (HLA) molecules. The HLA loci coding for these molecules are found in major histocompatibility complex (MHC) region, the most polymorphic region in the human genome, with over 15,000 HLA alleles observed so far in the world population. A high frequency of many different HLA alleles is thought be sustained by balancing selection. New HLA alleles may have an advantage over existing frequent alleles since immune escape mutations in pathogens within a population are maintained primarily in epitopes presented on frequent HLA alleles. Host immune function is not determined by single HLA alleles, but by both copies of autosomal HLA genes together (genotypes). Complementarity in function across the two potentially-variant copies of HLA at each locus can result in overdominance and heterozygote advantage at the genotype level. Less explored are selection mechanisms that may be operating across combinations of HLA alleles across loci (haplotypes). Indeed, in addition to high allelic diversity, HLA also has distinctive patterns of haplotype diversity, as certain HLA alleles co-occur in high linkage disequilibrium across five classical HLA loci (HLA-A, -B, -C, -DRB1, -DQB1). We applied multiple population genetic models to a dataset of HLA haplotype frequencies derived from over six million individuals with the goal of determining what type of selection may impact HLA haplotype diversity. We found frequent haplotypes were preferentially maintained in the population across 23 US populations studied. Thus, balancing selection at the allele level and purifying selection at the haplotype level may together affect HLA diversity in human populations.

## Introduction

Human leukocyte antigen (HLA) genes in the major histocompatibility complex (MHC) on Chromosome 6 provide the core function of antigen presentation for the adaptive immune system. Each HLA allele can present a restricted repertoire of peptides from either self or non-self proteins to T cell receptors. HLA loci are among the most polymorphic in the human genome [1] [2], as are their close MHC homologs in other organisms [3]. HLA allele homozygotes have been suggested to be at a significant disadvantage in that their peptide repertoire is more limited than heterozygous individuals [4]. Other genetic systems that have comparable levels of polymorphism to HLA in humans include the olfactory receptors and killer immunoglobulin-like receptors (KIRs) [5,6].

The direction and magnitude of selective pressure on genes can be estimated through the analysis of allelic variation within populations. For most genes, the nonsynonymous substitutions that alter the amino acid sequence of a protein are typically neutral or deleterious, with very few advantageous variants appearing. However, for HLA genes amino acid variation in the antigen recognition domain of HLA proteins determines the repertoire of peptides loaded onto the HLA protein and presented to the T cell receptor. Compared to other genes nonsynonymous variants in HLA genes were proposed to be often advantageous because a novel peptide repertoire may improve control of evolving pathogen strains [7]. Comparing patterns of variation among different genes, HLA has among the highest ratios of nonsynonymous substitutions relative to synonymous substitutions, which is a hallmark of balancing selection (i.e. selection maintaining a larger number of alleles than expected from genetic drift). Furthermore, the Ewens-Watterson test for neutrality also shows that observed HLA allele homozygosity is less than expected, another indicator consistent with balancing selection [8].

Host-pathogen co-evolution has also been proposed to lead to balancing selection that maintains high levels of HLA allelic diversity within populations. Viral escape mutations may be positively selected even if they have a fitness cost to the virus [9–13], reducing the number of epitopes from existing viral variants that can be presented by frequent HLA alleles. This process of immune evasion could produce a fitness advantage to hosts carrying rare HLA alleles. As pathogens maintain escape mutations in epitopes presented by the more frequent HLA alleles within a population, negative frequency-dependent selection favors less frequent HLA alleles [14].

While the forces shaping the HLA allele frequency distribution have been extensively discussed, the forces affecting co-occurrence of alleles across HLA loci and the resulting haplotype (allele combination) frequency distribution have not yet been thoroughly examined. HLA haplotype dynamics add another layer of complexity since HLA alleles are in clear linkage disequilibrium [15]. Some sets of HLA alleles co-occur on haplotypes more often than expected given the allele frequency distribution and other sets of alleles co-occur much less often than expected. A large number of new haplotypes emerges in the human population in every generation through recombination when compared to less frequently occurring mutation events. For example, the Southeast Asian population and the European populations have been estimated to diverge about 23,000 years ago [10], yet 64% of the Asian HLA haplotypes are not represented in the European population. HLA haplotype diversity across loci is thus far greater than the allelic diversity at a single locus.

Viral escape mutations typically alter recognition by a single HLA allele. However, specific haplotypes may provide better control of pathogens, or ensure proper activation of natural killer (NK) cells [16]. The innate immune response can be modulated by engagement of inhibitory KIR receptors on NK cells with HLA Class I ligands on target cells. The KIR ligand status is dependent on epitopes present on a subset of HLA alleles. Different HLA alleles will engage with different KIRs, or no KIRs at all, and these interactions influence the degree to which NK cells are licensed to kill target cells in which HLA expression is disrupted. Beyond immune modulation from HLA polymorphisms there is also genomic copy number variation for the number of inhibitory and activating KIR receptors.

Allele combinations at different loci on the same haplotype that present multiple epitopes from frequent pathogens could be preferred because they could have better redundancy to prevent immune escape. Interestingly, new results using the ratio of synonymous to non-synonymous mutations suggest selection favors heterozygotes with more divergent allele sequences [17]. This same mechanism preferring divergence in sequence (and therefore function) could also apply across HLA loci on haplotypes. Epistatic selection has been argued to affect immune and autoimmune responses [18]. Within the HLA locus, epistatic effects have been observed in the class II region [19]. In viruses, combination of epistasis and balancing selection has been shown to affect the genomes of viral populations [20].

While the high linkage disequilibrium (LD) between HLA alleles has long been known [21], possible models for the selection inducing this LD and its effect on haplotype frequency distribution have never been studied empirically. Such an analysis has been hampered by the need for extremely large samples to ascertain the shape of the haplotype frequency distribution. To meet this challenge we employed a large dataset of HLA haplotype frequency estimates for 23 United States populations derived from over six million volunteer stem cell donors recruited by the National Marrow Donor Program (NMDP) registry [22].

We developed a set of population genetic models to attempt to infer the selection pressures that operate on this large haplotype frequency distribution. We here present multiple pieces of evidence indicating that the HLA haplotype frequency distribution deviates from expectations under neutral evolution [23], and conclude that selection favoring existing frequent haplotypes best explains the distribution of HLA haplotypes observed.

## Results

### Validation of HLA haplotype frequency distributions

High resolution HLA haplotype frequencies were previously estimated using the expectation-maximization (EM) algorithm based on NMDP registry HLA genotypes [24]. Accuracy of HLA haplotype frequency estimates are limited by the resolution of the input genotypes [25]. HLA typing assays historically could not distinguish between all known HLA alleles, due to either lack of ability to phase polymorphisms within the gene, or lack of complete sequencing of the gene, or both. The resulting HLA typing results were considered “low resolution” and are represented by a list of possible allele pairs, or genotypes, that might be present at that locus. “High resolution” genotypes are generated when all known alleles that differ in the exons coding for the antigen recognition site (exons 2 and 3 for class I and exon 2 for class II) were distinguished experimentally. The NMDP haplotype frequencies were estimated at high resolution and utilized as input both low resolution genotypes and high resolution HLA genotypes. Low resolution typing within a locus can result in misidentification of the high resolution allele in the frequency data, while a lack of experimental data on haplotype phase between HLA loci can lead to the construction of incorrect arrangements of high resolution alleles into haplotypes.

**Fig 1:**
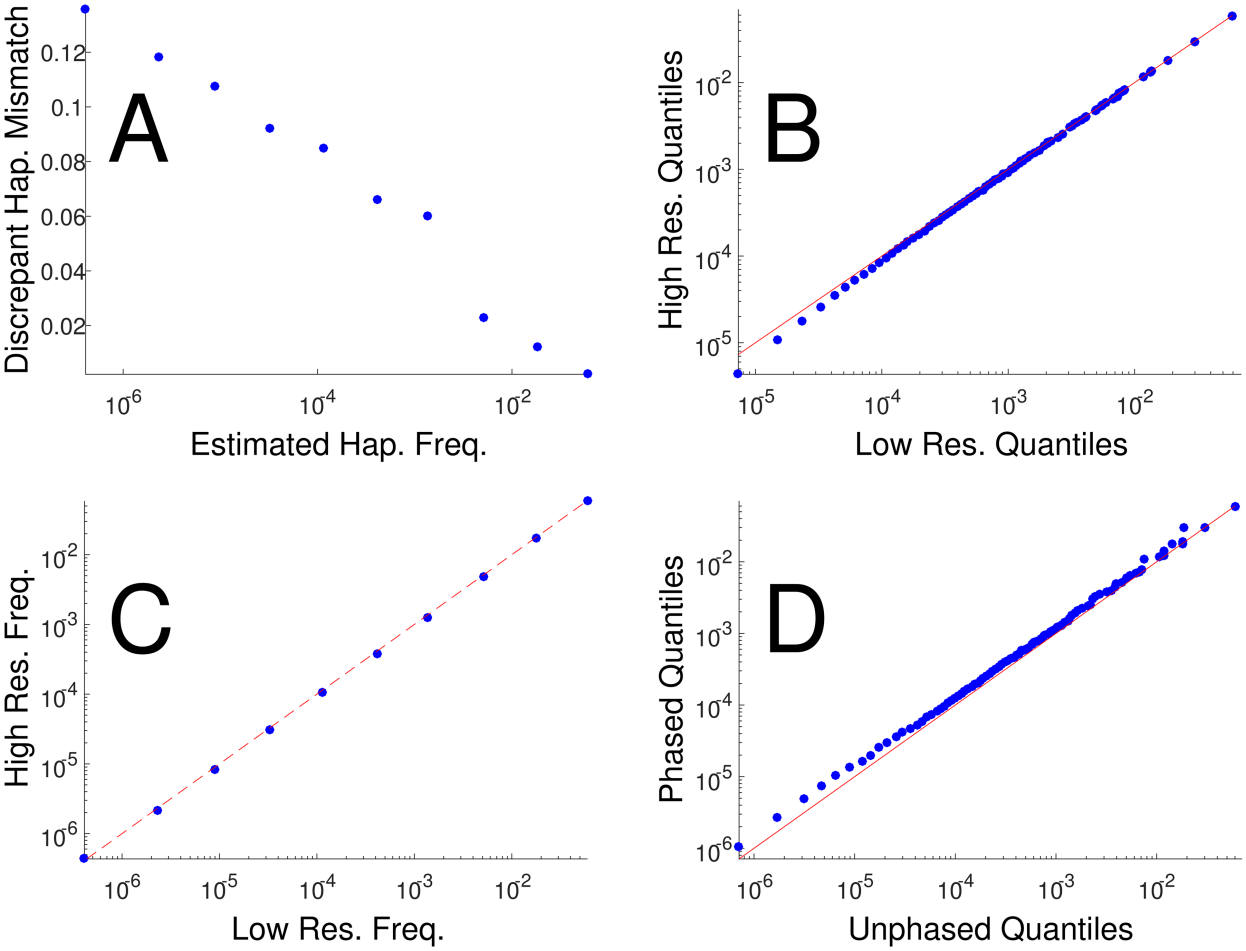
Methodology validation. (A)—Fraction of discordance between the computed most probable haplotype for an individual based on low resolution typing and the haplotype measured from high resolution typing. The discordance level is computed as a function of the low resolution typing haplotype frequency. (B) Quantile-Quantile (QQ) comparison of the frequency distribution of the most probable haplotype per individual as computed from low resolution typing and the parallel from high resolution typing. The distribution was computed over all donors with both high and low resolution typing. The x-axis is the frequency of a haplotype at the kth quantile in the low resolution based EM estimate, and the y-axis is the parallel in the high resolution typing. Values on the diagonal imply a similar cumulative distribution function (CDF) (C) The observed haplotypes were logarithmically binned by their low-res frequency and the geometric means of both the low and high resolution frequencies were calculated, resulting in largely the same values for each bin. (D) Quantile-Quantile comparison of the haplotype distribution in the same patients between phased and un-phased genotypes.

In order to assure that EM-based estimates of HLA haplotype frequency distributions are suitable for estimating selection, we performed several validation procedures. To assess the effect of incomplete HLA typing, we analyzed 38,715 donors with both high and low resolution typing and measured concordance between the predicted high resolution haplotype constructed from the EM algorithm using the low resolution typing and the experimentally measured high resolution typing (Fig 1A). The discordance rate (most probable predicted high-resolution haplotype different than actual) varied from 14% for very rare haplotypes (frequency<1.e-7) to less than 1% for frequent haplotypes (f>1.e-2). However, this discordance does not lead to an error in the overall shape of the frequency distribution, since low frequency haplotypes are typically replaced by other low frequency haplotypes and not by high frequency haplotypes, as can be seen by the Quantile-Quantile (QQ) plot (position of percentiles of one distribution vs the other) of the low and high resolution haplotype distributions (Fig 1B). To further validate that the correct haplotypes are replaced by discordant haplotypes of similar frequency, we divided all haplotypes based on their frequency in the EM based haplotype distribution. We then computed the average frequency of the appropriate high resolution haplotypes. As can be seen in Fig 1C, the distributions are similar.

To validate that phasing errors do not affect the shape of the expected haplotype distribution, we compared haplotype phasing of 4,000 cord-mother pairs using EM versus direct counting using pedigree analysis (see Methods). We found that the shape of the haplotype frequency distribution was not appreciably affected by EM phasing errors. (Fig 1D).

### Selection models for alleles and haplotypes

In order to correlate haplotype and allele frequencies with possible selection models, we define here in detail the terms we use describing selection forces for alleles and haplotypes along with the many underlying evolutionary mechanisms that have been proposed to contribute to these selection forces.

We use “balancing selection” as an umbrella term for all those selection pressures that lead to a greater diversity in HLA allele frequency distributions than what would be expected under a neutral evolutionary model.

Underlying this balancing selection are several distinct evolutionary mechanisms that together may combine to form the allele frequency distributions we observe. The model of negative frequency-dependent selection, or rare allele advantage, suggests that continual evolution of viral strains to evade common HLA variants maintains high diversity in HLA alleles in a population. Heterozygote advantage, or overdominance, is a model where heterozygote genotypes have higher fitness than homozygote genotypes. Heterozygotes are capable of presenting a wider peptide repertoire than homozygotes, which would confer improved likelihood of immune detection of pathogens. Takahata and Nei found that “Minority advantage considered here produces essentially the same pattern of genetic polymorphism as that for overdominant selection”, and many other researchers have since encountered similar challenges in teasing apart the mechanisms behind balancing selection [26].

We here introduce "purifying selection" as a parallel umbrella term for all those selection pressures that lead to less diversity in HLA haplotype frequency distributions than what would be expected under a neutral model.

Several evolutionary mechanisms may underlie purifying selection. Under purifying selection, rare deleterious mutations are continually purged from populations because they contribute to lower fitness. Purifying selection can occur at the haploid level as HLA alleles are co-dominant or at the diploid level in the case of recessive deleterious mutations in the MHC. Positive frequency-dependent selection is another potential mechanism that would favor more frequent HLA haplotypes that most effectively modulate the immune response. As was the case with alleles, both frequency-dependent and non-frequency dependent mechanisms can induce the same haplotype frequency distribution we observe.

### HLA allele frequencies show signs of balancing selection

HLA allele frequencies have long been shown to be consistent with balancing selection, [8,15]. An important indicator for the action of balancing selection is the Ewens-Watterson homozygosity test [27]. In order to test for balancing selection on single-locus allele distributions within the studied populations, we performed Ewens-Watterson tests on random subsamples of 1,200 alleles for each US subpopulation [28] (S1 Table) We also computed the observed and expected homozygosity (as predicted from the sum of squares of haplotype frequencies), and normalized deviate of homozygosity *Fnd* [29] of the subsamples. Negative *Fnd* indicates observed homozygosity below expected homozygosity.

**Fig 2:**
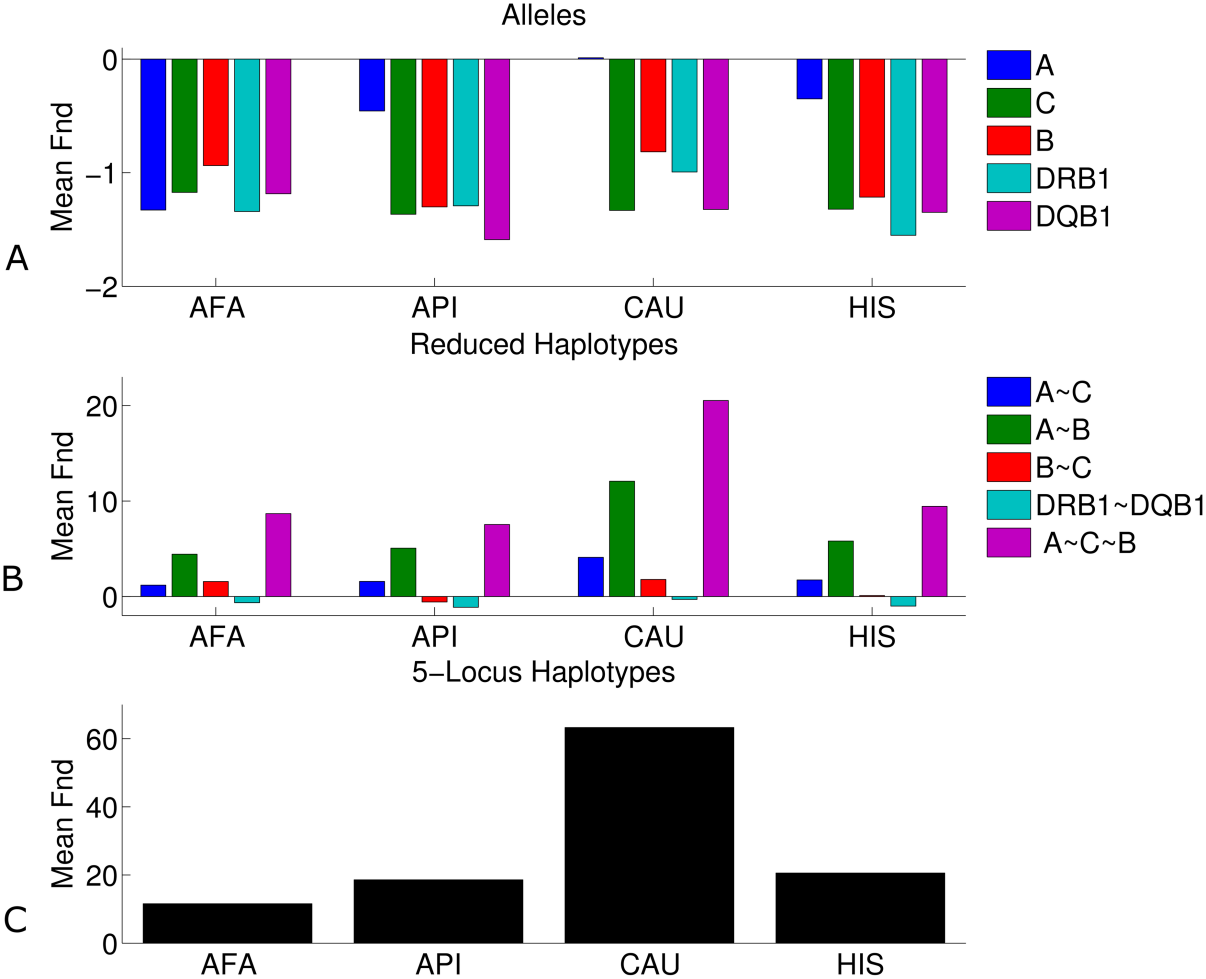
Fnd values of haplotypes and allele frequencies. The difference between expected and observed homozygosity, as defined by the Fnd of alleles (A plot), haplotypes (C plot) and allele combination (B plot) frequency distributions, calculated from subsamples of 1,200 individuals in 4 broad race groups (AFA—African American, API—Asian and Pacific Islander, CAU—European (Caucasian) and HIS—Hispanic). Single-locus Fnd values are negative (lower observed than expected homozygosity) in most populations, indicating that observed homozygosity exceeds that expected for a constant-size neutrally evolving population (upper plot). Contrary to the single-locus results, Fnd values of full 5-locus haplotypes are positive, denoting multi-locus homozygosity above expectation (C plot). This extra homozygosity holds most strongly for Class I loci, versus Class II loci. The Fnd of two-locus Class I haplotypes containing the HLA-A locus show a positive Fnd value (B plot).

The Ewens-Watterson test shows that many, but not all, populations exhibit homozygosity values significantly lower than the expectation from neutral evolution in a fixed population, suggesting balancing selection (See Methods for multiple measurement corrections method). Further, negative *Fnd* values were observed for the HLA alleles in all subpopulations (Fig 2 and S1 Appendix showing lower homozygosity than expected in alleles) in agreement with previous reports [8]. The largest difference between expected and observed homozygosity occurred in HLA-C and DQB1(average *Fnd* values of -1.23 and -1.33), and on average over all populations, balancing selection was observed in all loci. Some populations show balancing selection more clearly than others, with Koreans and Caribbean Hispanics displaying the strongest deviation from neutrality. The only notable exception to this observation is the DRB1 locus in the Filipino population, which did not exhibit balancing selection.

Detecting selection on haplotypes is more complex than for alleles. Thus, we applied a set of different tests—all showing clear signs of deviation from neutral drift toward a lower than expected diversity, consistent with models, where existing frequent haplotypes are favored over new rare haplotypes.

### Linkage disequilibrium patterns shows more frequent haplotypes than expected in hardy-weinberg equilibrium

We computed the normalized linkage disequilibrium value *D*_*ij*_' for all 2-locus HLA haplotypes, as defined by Lewontin [30], and estimated its value as a function of the haplotype frequency. Lewontin’s *D*_*ij*_' is a normalized coefficient of linkage disequilibrium between two specific alleles ranging between -1 and 1, with positive values indicating that the specific 2-locus haplotype is more frequent than expected by the marginal frequencies of the two alleles. A 2-locus haplotype is defined as a combination of two alleles (e.g. one allele of HLA-B and one allele of HLA-C) or two haplotypes of multiple loci in the same class cluster (one haplotype of Class I and one haplotype of Class II where each haplotype is treated as if it were a single-locus allele). In a neutral model, rare haplotypes would have a low *D*_*ij*_ value, while frequent haplotype would have a high *D*_*ij*_ (See S2 Text for simulation results). Positive FDS or similar purifying selection mechanisms would push more haplotypes from intermediate to high frequency values (for selectively favored haplotypes) and to low values (for haplotypes selected against). Positive FDS may thus produce negative *D*_*ij*_' values for intermediate frequencies and positive values for low and high frequencies.

**Fig 3:**
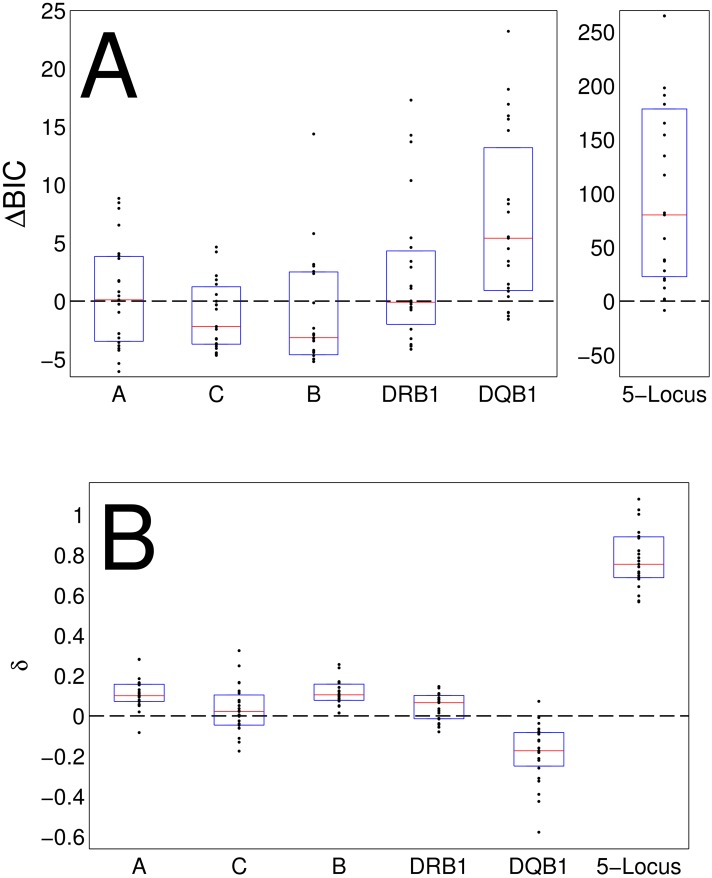
Linkage disequilibrium patterns. Mean normalized *D*_*ij*_' (using the Lewontin normalization) as a function of the frequency for four broad race populations.*D*_*ij*_ is a normalized measure of linkage disequilibrium (LD) taking values between -1 and 1 (maximal LD) and in neutrality should be 0. Each allele/haplotype-pair was assigned its combined frequency and a *D*_*ij*_' value and the *D*_*ij*_' values were averaged over all allele pairs within the same frequency bin in a given population. One can observe that for all allele pairs, the average *D*_*ij*_' values are zero for very low frequencies, null or negative for intermediate frequencies and highly positive for high frequencies. This pattern of *D*_*ij*_' values indicates that frequent allele pairs are much more frequent than expected by the frequencies of their components (e.g. p(AB>P(A)P(B) for high P(AB) values, but P(AB)<P(A)P(B) for intermediate P(AB) values).

An analysis of the patterns of linkage disequilibrium in all four relevant pairs of loci: A~B, A~C, B~C, and DRB1~DQB1, across all populations uniformly shows this bimodal pattern with *D*_*ij*_' of rare haplotypes close to zero, negative linkage disequilibrium for intermediate frequency haplotypes, and positive LD for the most common haplotypes (Fig 3). The main effect was observed for the A~B and A~C Class I haplotypes, in agreement with the results from the Ewens-Watterson tests discussed below.

In order to test that this linkage disequilibrium pattern is not an artifact of the population dynamics, we have performed simulations of neutral evolution (See S1 Text for description of simulations), using realistic parameters for the HLA loci (See S2 Table for parameter estimates), and show that the neutral simulations do not produce the observed LD patterns. (S2 Text. Section 1). These simulations were performed for either constant or growing populations and with or without population substructure. We have further produced model simulations of populations with positive FDS to show that such populations can display a minimal value of *D*_*ij*_ in intermediate frequency ranges (S2 Text Section 3). This is obviously not a proof that positive FDS is the model driving the observed dynamics, only that positive FDS is a possible mechanism for the purifying selection of HLA haplotypes.

### Ewens-Watterson test on haplotype frequencies show excess homozygosity

In order to further test deviation from the null model of neutral evolution, we performed the Ewens-Watterson test on the five-locus haplotype frequency distribution. In contrast with allele frequency, in haplotype frequency distributions a clear positive and significant *Fnd* is observed for the 5-locus haplotype frequencies of all populations (average Fnd = 28.43). The resulting positive *Fnd* values are consistent with positive selection. (Fig 2 and S1 Appendix). These results are robust to sampling and to changes in sample size (S2 Text Section 4). While the Ewens-Watterson test was developed specifically for non-recombining loci, its efficacy in the detection of positive selection in the case of haplotypes has previously been established [31]. Moreover, the deviation from neutrality attributed to recombination generally decrease the haplotype homozygosity [32,33] and is thus not expected to be interpreted as positive selection. In order to test that the presented results are not the result of recent population growth, population substructure, the high recombination and mutation rate of the HLA loci, the balancing selection on alleles or sampling effect, we simulated such scenarios (see detailed list of scenarios studied in S1 Text). None of the simulated scenarios produced deviation from neutrality that approached what was observed in our haplotype frequency distributions (S2 Text Section 5). Moreover, none of these scenarios led to a combination of positive Fnd values for haplotypes and negative *Fnd* values for alleles. However, when balancing selection on alleles is combined with purifying selection on haplotypes the opposite deviations from neutrality can be easily obtained (S2 Text Section 6).

In order to test the robustness of these results to sampling, we have repeated the analysis 100 times for each subpopulation, and obtained a very limited variance over all populations studied (S2 Text Section 5). As was the case for alleles, large variation in *Fnd* values was observed among all populations. Among the broad race groups, the *Fnd* statistic was significant at the p<0.001 level after multiple measurement corrections for all populations. *Fnd* was largest in the European population, and comparably large for the rest, while among the detailed race groups the Vietnamese population showed the strongest effect (S1 Appendix). Note that all populations show a very clear deviation of excess homozygosity, in opposition to the observation in the allele frequency distributions.

### Positive *Fnd* is restricted to HLA Class I

In order to test which haplotypes affect the deviation from the null hypothesis of neutral evolution, we performed the *Fnd* test described above on two-locus and three-locus HLA Class I and two-locus Class II haplotypes. The results show positive *Fnd* across most populations for the HLA Class I haplotypes, and especially for haplotypes containing the HLA-A locus. Meanwhile, *Fnd* values were mostly negative for the DRB1~DQB1 HLA Class II haplotypes (Fig 2).

#### Excess homozygosity is not caused by population substructure

Excess homozygosity is often observed in HLA population samples that contain a mix of genetically-distinct populations. This phenomenon has been termed the Wahlund effect [34]. To rule out the confounding impact of any population substructure in our data on our selection model results, we present the following lines of evidence:

- We observed a clear difference between the measures of selection on different sets of loci in the same population. Differences in *Fnd* values were observed between class I haplotypes and class II haplotypes. Such differences, on the same set of individuals, cannot be explained by population substructure.
- The more divergent and homogenous populations, such as the Japanese population, show a clear deviation from neutrality in the direction of purifying selection, just as the admixed populations do (Mexican, African American).
- Simulations with substructure do not show opposite deviations in homozygosity for single loci versus haplotypes.

### Fitting evolutionary models for deviation from neutrality to HLA haplotype frequency distributions

The *Fnd* statistic and p-values from the Ewens-Watterson test are measures of deviation from neutrality and equilibrium and not directly measures of selection. Other factors may affect the observed deviations, as has been explored by Akey et al [35]. The *D*_*ij*_' measure may also be affected, in theory, by other elements that impact LD. In order to directly test whether purifying selection models are better consistent with the observed distribution than neutral models, we directly analyzed the haplotype frequency distribution—examining the relation between the frequency of a haplotype in the sample and the number of unique haplotypes at that frequency.

By very general arguments, one may describe the allele and haplotype frequencies by a birth-death process. If birth and death are balanced, the population can be studied in equilibrium, while if birth exceeds death, an out-of-equilibrium model must be constructed. If one assumes no selection, two models can be considered:

1. The marginal distribution of Ewens' sampling formula, which assumes a constant population size and neutral evolution [36] composed of birth, death and random mutations.
2. Yule-Simon distribution, which results from a process of pure birth, exponential growth and neutral evolution [37]. The difference between the Yule model and the Ewens distribution is that death is neglected, and the population grows exponentially.

One can model purifying and balancing selection through a frequency-dependent selection process. If such a model is invoked, a third type of stochastic model known as a Birth, Death and Innovation Model (BDIM) can be used to fit the observed frequency distribution. BDIM models admit the possibility of density-dependent growth and death rates, which can be interpreted as a non-neutral evolution [38]. Specifically, the total growth and death rates of each sub population are proportional to the population size plus a constant. If these constants are 0, the model is neutral. The details of all models are explained in S2 Text Section 7. Note that in this context density-dependent selection can be used as a rough proxy for other types of selection in the sense that the resulting haplotype frequency distribution can be compared with the one expected in neutral evolution.

In order to identify which model best represents the observed distributions, we fit both the Yule-Simon and BDIM models to the frequency distributions of haplotypes using a maximum likelihood approach. The Ewens model was not suitable for fitting because it has no free parameter (except for a normalization constant), and the distribution did not fit the observed distribution. The functional forms of all distributions are described in the methods section. The BDIM model contains a selection parameter, which determines whether small populations have a higher or lower net growth rate than large populations. A positive value implies that small populations are selected against (positive FDS favoring existing frequent haplotypes), while a negative value implies that small populations are selected for (balancing selection).

**Fig 4:**
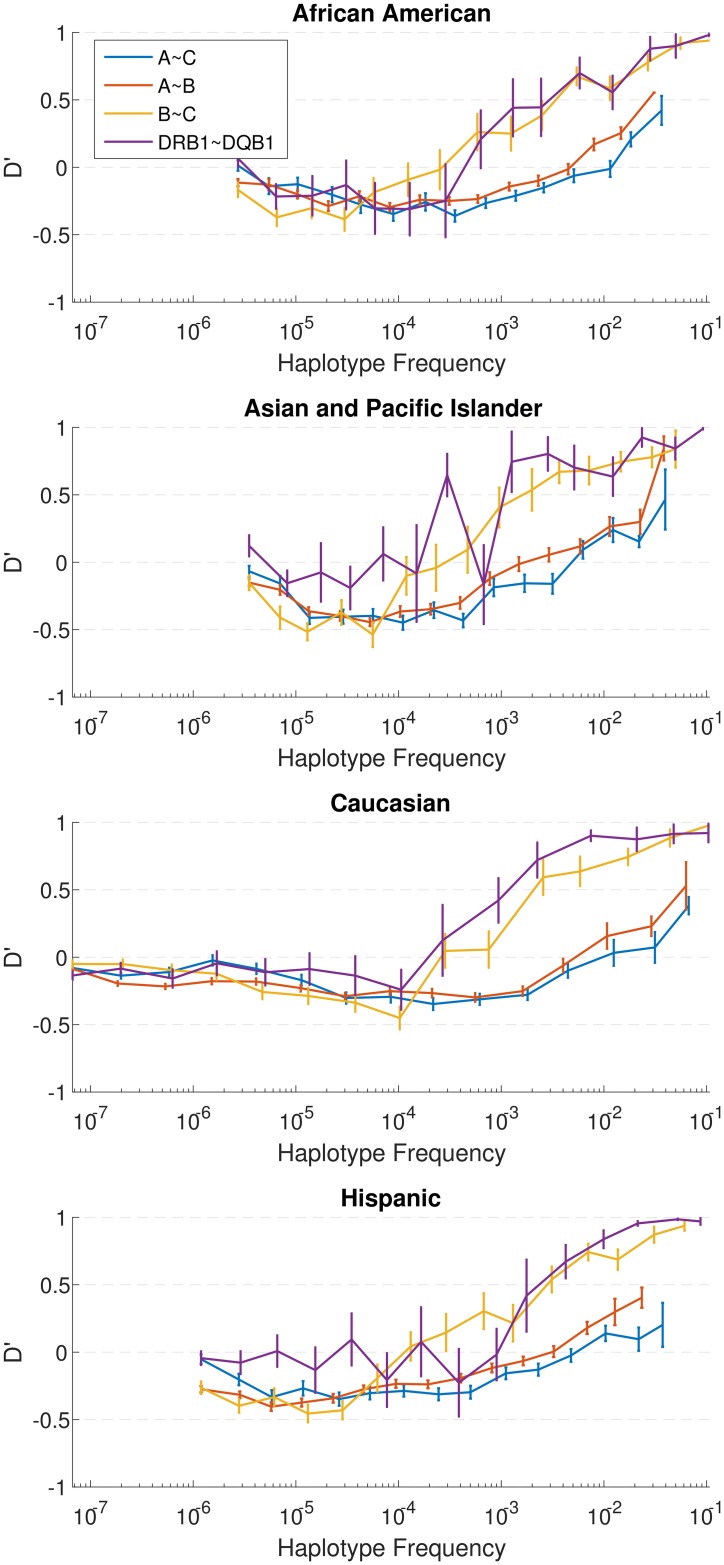
Comparison of frequency models. **(A)** The difference between Bayesian Information Criterion (BIC) values of the maximum likelihood estimate (MLE) fit of the Yule model and the BDIM. The models were fitted to frequency distributions of each allele separately, as well as to the full 5-locus haplotype frequency distribution. The calculation was repeated for all 18 detailed race groups and 5 broad race groups (black dots). The red line represents the median over the 23 populations, while the blue lines represent the other quantiles. **(B)** The selection parameter *δ* of the MLE fit to the BDIM. This parameter represents the net reproductive disadvantage of the rare frequencies. Thus, positive values suggest a disadvantage to rare alleles/haplotypes.

Model selection was performed using the Bayesian Information Criterion (BIC) measure which incorporates both quality of fit and number of parameters—more parameters have a higher penalty to avoid overfitting. Among the models above, the BDIM model had a significantly better fit with the HLA haplotype frequency distributions, even when accounting for its extra parameter by using BIC for model selection [39] (Fig 4A). The positive ΔBIC values for BDIM minus Yule (which is better than Ewens) indicate that BDIM is produces a significantly better model than any of the neutral models. The selection parameters in the BDIM model, which can be interpreted as the net fitness disadvantage of rare haplotypes, were significantly larger than 0 (Fig 4B). No significant advantage existed for either model in fitting the allele frequency distributions. In order to estimate absolute fit of the BDIM model to the data, the allele and haplotype frequency distribution was binned into 20 logarithmic bins, and the R^2 value of the comparison between the predicted and observed distribution was computed for all populations and all allele and allele combinations, as in Fig 4. The average coefficient of determination (R^2) value over populations, alleles and haplotypes (A, B, C, DR, DQ and 5 locus haplotypes)was 0.75. and 0.68 for the Yule model.

## Discussion

Applying several population genetic models, we find that frequent HLA haplotypes occur more often than would be expected under a neutral evolutionary model in all studied US populations, which suggests purifying selection. At the same time, we corroborated numerous previous studies showing that balancing selection may be operating at each individual HLA locus. Taken together, the multiple complementary analyses, fitting the frequency distributions to different evolutionary models, the *Fnd* measure of homozygosity deviation from a null model, and linkage disequilibrium analysis, all suggest purifying selection at the haplotype level, and that positive FDS provides a good fit for the haplotype frequency distribution. While we do not explicitly simulate the diploid fitness-based selection models, theoretically both frequency-dependent and non-frequency-dependent mechanisms could produce the same type of distribution.

To our knowledge, this is the first time that explicit evolutionary population dynamics models have been compared across such diverse populations at a scale of millions of individuals. While these models utilize the mechanism of frequency-dependent selection, they may be a proxy for other purifying selection mechanisms. Indeed, many other researchers have found that multiple disparate evolutionary mechanisms each capable of producing the same shape for observed frequency distributions [40]. While we are unable to tease apart the exact mechanisms involved, the main contribution of this paper is the identification of empirical data suggesting that selection has an opposite impact on allele frequency versus haplotype frequency distributions. Note that we have not explicitly modeled population structures. We will now develop models combining population structure and selection to test their combined effect.

Multiple previous results suggest that HLA haplotype frequencies are shaped by selection. The high levels of linkage disequilibrium observed among HLA alleles serve to limit the amount of diversity in HLA haplotypes and multi-locus genotypes. Several different HLA haplotypes have been maintained at high frequency in different populations over long periods of time and have been termed “conserved extended haplotypes” or “ancestral haplotypes” [41]. If the HLA system had lower linkage disequilibrium, more combinations of alleles at different loci would be observed at higher frequency in populations. Finally, the amount of genetic recombination between HLA loci does not correlate directly with genetic distance in the MHC [42], indicating that selection may be shaping patterns of human MHC haplotype variation.

As the recombination rate between HLA loci is faster than the allele formation rate through mutation or gene conversion, these two processes can be interpreted as two different time scales in our evolutionary models. Over long time scales, new alleles are introduced within a single locus. At shorter time scales, certain haplotypes are generated from existing alleles by recombination. While new haplotypes continuously arise, the number of highly successful haplotypes would be limited compared to the space of all possible combinations. These successful conserved extended haplotypes may be maintained in populations. The fitness of specific haplotypes and multi-locus genotypes thereof may differ over time and among populations [14].

Within the HLA region, the most significant deviation from neutrality was observed in haplotypes composed of Class I haplotypes, while no such deviation was observed in the Class II haplotypes. This evidence for Class I selection is in good agreement with the effect of Class I variation on survival in the presence of different pathogens [43,44], and also may be correlated with the interaction between HLA Class I and KIR [45]. Note that the functions of Class I and Class II alleles and haplotypes can have epistatic effects that may impact how selection operates on the overall HLA system. Moreover, differences in migration rate in class I and class II could explain the difference between the two regions, However, such differences cannot explain the difference between alleles and haplotypes.

A possible caveat is the sample used. In theory, the NMDP cohort may not be representative of the general population. However there are no obvious recruitment practices that would lead significant systematic HLA genetic bias within donor populations. Thus it is common practice for registry and blood bank donors to be used as controls in disease association studies (e.g. the Wellcome Trust Case Control Consortium used blood bank donors). Moreover, there are no obvious reasons that such a misrepresentation would affect the difference between alleles and haplotypes.

Multiple evolutionary mechanisms may explain purifying selection for HLA Class I haplotypes. HLA alleles found along the same haplotype may have complementary peptide repertoires across loci to present multiple epitopes from a single viral protein simultaneously. In the case of SIV in monkeys, there has been selection for certain combinations of HLA Class I alleles across loci that control SIV and its escape variants [46]. If pathogens require multiple mutations to achieve immune escape from all HLA alleles in an individual, the likelihood of escape is minimized. Haplotypes containing alleles with redundant recognition capabilities may be preferentially selected for fitness in individuals, while haplotypes without complementary repertoires would be eliminated. Alternatively, haplotypes may need to present epitopes from multiple different viruses. We have shown that the number of epitopes presented by different HLA alleles can vary over many orders of magnitude [11,47]. Haplotypes with more limited epitope repertoires may be detrimental, and selected against. Finally, as mentioned, interactions with KIR proteins on natural killer cells may determine the capacity of the immune system to mount a response [48], requiring specific HLA allele combinations to ensure adequate response.

A possible unified selection model for the patterns of diversity observed in both HLA alleles and haplotypes has been proposed by van Oosterhout called Associative Balancing Complex (ABC) selection provides an explanation for how linkage disequilibrium between HLA alleles could be maintained by epistasis in the MHC region [49]. Under this model mutations in MHC haplotype blocks accumulate under a sheltered load near HLA genes. Recombination in HLA haplotypes would expose low fitness homozygous genotypes. Epistatic selection operates against this recombination and increase linkage disequilibrium. Purifying selection against deleterious recessive mutations is weak because recombination is low. Frequent HLA haplotypes are maintained and increase divergence from one another over time. While we do not model ABC selection explicitly, our data is consistent with this model of balancing selection on HLA alleles, epistatic selection that limits recombination, and purifying selection in HLA haplotypes.

HLA genes are distributed throughout the MHC throughout a large ~4-megabase region of Chromosome 6. Because the distance between HLA loci can be as much as 1 megabase, HLA haplotype phase cannot be experimentally determined with current classic HLA typing methods. HLA alleles were phased into haplotypes computationally using the expectation-maximization (EM) algorithm rather than experimentally. The EM algorithm attempts to find a maximum likelihood estimate wherein all HLA unphased genotypes are explained using a minimal set of HLA haplotypes. We have here shown that while the EM produces a non-negligible level of allele classification and phasing errors, these errors have a minimal effect on the shape of the resulting haplotype frequency distribution. Population substructure in HLA-typed cohorts can cause excess homozygosity which would confound the selection model results, however we find that our consistent results across populations, along with differing forces at Class I versus Class II rules out the possibility that population substructure could explain our findings. The HLA-DP locus has not been included in our analysis due to the historical absence of typing information in the registry and subsequent haplotype frequency datasets. We recognize that extending this analysis to include this locus will be challenging due to the relatively higher rate of recombination between HLA-DP and the other HLA loci in this study [50–52].

The here described opposite selection types and alleles and haplotype levels may be a general evolutionary principle combining the introduction and novelty and the maintenance of high fitness combination. We now plan to further study theoretically and experimentally the presence of such mechanisms in other genomic regions and organisms.

## Methods

### Population haplotype frequency estimates

Five-locus high resolution HLA A~C~B~DRB1~DQB1 haplotype frequencies were estimated using the expectation-maximization (EM) algorithm for over six million donor HLA typings from the National Marrow Donor Program registry (USA) published by Gragert et al [14,53]. Given typing ambiguities, a large number of very low probability haplotypes emerge from the EM. The frequency distribution of such rare haplotypes and genotypes is a mere artifact of the EM. We removed low probability haplotypes by assigning each person in the sample a single pair of their most probable haplotypes with the remainder of their haplotype pair probability distribution discarded. The population haplotype frequencies were thus recalculated with a single haplotype pair assigned for each individual. Allele frequencies were derived as marginal sums of the haplotype frequencies.

In order to confirm the accuracy of these haplotype frequency estimates, we performed two validation experiments in which the most likely high resolution HLA genotype was imputed for individuals that had HLA typing ambiguity [25]. The first validation dataset, intended to confirm the accuracy of high resolution HLA allele identification, consisted of 38,715 registry donors who had high resolution confirmatory typing performed on behalf of a patient, allowing for comparison of the imputed high resolution allele with the true high resolution allele determined experimentally. The second validation set, intended to confirm accuracy in haplotype phase assignment, consisted of a cohort of 4,235 cord blood units where the cord mother was also HLA typed, and therefore haplotype phase was known by pedigree and could be compared with the results of imputation.

We computed frequency distributions for two-locus and three-locus haplotypes by summing over the frequencies at the other loci (i.e. produce the marginal distributions). For example, to compute the two-locus A~B haplotype frequencies, we merged all extended A~C~B~DRB1~DQB1 haplotypes with a given A~B combination into a single reduced A~B haplotype. We computed haplotype distributions for five different combinations of loci (A~B, A~C, B~C, A~B~C, DRB1~DQB1) in this manner.

### Ewens-Watterson test for deviation from neutral evolution

In the Ewens-Watterson test, one calculates the hypothetical F1 homozygosity following random mating (the sum of squared allele frequencies) of the sample, and compares it to the expected homozygosity of a sample with the same attributes (sample size and total number of alleles) from the Ewens sampling formula. The expected homozygosity and the p-value of the test are usually obtained using Slatkin's method [54]. The test parameters are limited by the numerical calculation of Ewens' sampling formula so that the maximal number of alleles in a sample is currently limited to a thousand and the maximal sample size to a few thousand. In order to perform such tests for extremely large samples such as ours, a representative subsample must be taken.

We randomly sampled 1,200 haplotypes from each US subpopulation and performed the EW test on the subsample, using either the Arlequin [55] or PyPop [56] software packages with similar results. We calculated *Fnd values* for each sample (*Fnd* = (*Fobs* − *Fexp*)/*Var(Fexp)*^1/2^), where *Fobs* is the observed homozygosity of the sample, and *Fexp* is the expected homozygosity for a population with the same parameters calculated using random samples, which also provided an estimate for the variance.

### Evolutionary models for frequency distributions

We fit the Yule model and a Birth, Death and Innovation Model (BDIM) detailed in Table 1 to the relative frequency distributions of haplotype/alleles populations by maximum-likelihood estimation, using a global optimization algorithm for the numerical maximization [57]. The normalizing constant *C* was determined by the equality:$C=\sum_{i=1}^{N_{\text{max}}}p(i)$, where *N*_**max**_ is the absolute frequency of the most abundant haplotype. This normalization is equivalent to fitting the model conditioned on the event that the maximal number of haplotypes is *N*_**max**_.

**Table 1:** 

Model name	probability function *p*(*x*)	Parameters	Comments	Reference
Yule	*C***B**(*x*, 1 + *v*)	*v*	C is a constant and **B** is the beta function.	[37]
BDIM	$C\frac{\Gamma (x+a)}{\Gamma (x+1+b)}$	*a*, *b*	C is a constant and Γ is the Gamma function. In Fig 4,: *δ = b* − *a*	[38]

### Linkage disequilibrium computation

We computed the linkage disequilibrium using the normalized approach proposed by Lewontin [58]. In short, the value of *D*_*ij*_' for each pair of alleles is normalized by the theoretical maximum for the observed allele frequencies. The *D*_*ij*_' value was computed for each pair of alleles *i* and *j* (e.g. given HLA A and HLA-B alleles), and binned across all haplotypes with similar frequency. Note that in this context a haplotype is either treated as a pair of alleles, or as a pair of haplotypes treated as alleles (e.g. a class I haplotype and a class II haplotype).

### Statistical analysis

A two-sided T-Test was used for comparing *Fnd* values and *D*_*ij*_' values for each population size bin to the neutral drift, and also testing evolutionary model fit. For the *Fnd* values, we treated each sample as an independent observation. In the *D*_*ij*_' analysis, each haplotype was treated as an independent sample. For the evolutionary model fit, each population was treated as an independent sample. Where relevant (e.g. where values were computed separately for each population) a Benjamini—Hochberg procedure to adjust for multiple tests using false discovery rate (FDR) was performed.

## Supporting information

S1 Table Population sample sizes. (DOCX)

S2 Table Estimates of recombination and mutation rates for HLA haplotypes. (DOCX)

S1 Text Description of simulations. (DOCX)

S2 Text Comparison of observed results with theoretical models. (DOCX)

S1 Appendix Ewens-Watterson test results for single alleles and haplotypes. (DOCX)

S2 Appendix Haplotype frequency files for all major US sub-populations. (ZIP)
