# Supplementary material for: HLA class I haplotype diversity is consistent with selection for frequent existing haplotypes
Source: PLoS Comput Biol. 2017 Aug 28;13(8):e1005693. doi: 10.1371/journal.pcbi.1005693 (PMC5590998; doi:10.1371/journal.pcbi.1005693)
Supplement: S1 Table — (DOCX) [file pcbi.1005693.s001.docx]

# S1 Table. Population Sample Sizes

18 detailed race/ethnic sub-populations and 5 broad race/ethnic populations were studied. Each category was defined based on the registry donor self-identified race and ethnicity (SIRE). Broad categories are marked in bold and represent the sum of the detailed categories listed above each broad category in table. Some populations are merged populations, and we mark the merged population associated with each sub-population.

| Symbol | Race Group | Sample Size | Global population |
| --- | --- | --- | --- |
| AAFA | African American | 1184776 | AFA |
| AFB | African | 77984 | AFA |
| CARB | Black Caribbean | 90538 | AFA |
| SCSEAI | South Asian | 507364 | API |
| FILII | Filipino | 144044 | API |
| HAWI | Hawaiian or other Pacific Islander | 36252 | API |
| JAPI | Japanese | 90340 | API |
| KORI | Korean | 208560 | API |
| NCHI | Chinese | 290480 | API |
| AINDI | Other Southeast Asian | 110040 | API |
| VIET | Vietnamese | 113748 | API |
| EURCAU | European Caucasian | 3472992 | CAU |
| MENAFC | MidEast/No. Coast of Africa | 198752 | CAU |
| MSWHIS | Mexican or Chicano | 716492 | HIS |
| SCAHIS | South/Cntrl Amer. Hisp. | 425480 | HIS |
| CARHIS | Caribbean Hispanic | 332920 | HIS |
| CARIBI | Caribbean Indian | 42484 | NAM |
| AMIND | North American Indian | 109796 | NAM |
| AFA | **African American*** | 1353298 |  |
| API | **Asian and Pacific Islander*** | 993464 |  |
| CAU | **Caucasian*** | 3671744 |  |
| HIS | **Hipanic*** | 1141972 |  |
| NAM | **Native American Indian*** | 152280 |  |
