# Supplementary material for: HLA class I haplotype diversity is consistent with selection for frequent existing haplotypes
Source: PLoS Comput Biol. 2017 Aug 28;13(8):e1005693. doi: 10.1371/journal.pcbi.1005693 (PMC5590998; doi:10.1371/journal.pcbi.1005693)
Supplement: S2 Table — (DOCX) [file pcbi.1005693.s002.docx]

# S2 Table. Estimates of recombination and mutation rates for HLA haplotypes

The human Major Histocompatibility Complex (MHC) region is located on the short arm of chromosome 6. Classical HLA genes reside in the MHC and are split into two classes, HLA Class I (containing A, B, and C loci) and HLA Class II (containing DRB1, DQB1, and DPB1 loci) ([MHC_sequencing_consortium 1999](#_ENREF_4), [Yeager and Hughes 1999](#_ENREF_7)). In order to estimate the HLA haplotype recombination rate in humans, we used the position of each gene in the chromosome and estimate of recombination rates in human chromosome ([Turpeinen, Volin et al. 2009](#_ENREF_6)). There is no unequivocal data measuring the recombination rate with the HLA region in the literature ([Cullen, Noble et al. 1997](#_ENREF_1), [Cullen, Perfetto et al. 2002](#_ENREF_2)), we thus estimate it from the base pair distances in the human genome. The distance between each HLA locus was defined as the difference between the last nucleotide of one locus and the first nucleotide of the second locus (See table below).

|  | A locus | C locus | B locus | DRB1 locus | DQB1 locus |
| --- | --- | --- | --- | --- | --- |
| First nucleotide | 29942470 | 31268749 | 31353868 | 32578769 | 32659464 |
| Last nucleotide | 29945884 | 31272136 | 31357212 | 32589836 | 32155578 |
| Distance between this locus and the next (Mbp) | 1.32 | 0.08 | 1.22 | 0.069 |  |

The range of recombination rates in chromosome 6 in the human genome is from 0.0111 events per Mbp and 0.0044 recombination events per Mbp across the HLA region on average ([Turpeinen, Volin et al. 2009](#_ENREF_6))The minimal recombination rate can be estimated as the product of the minimal rate for recombination 0.0044 by the smallest distance between the loci 0.069 Mbp = 3*10^-4^.

The maximal recombination rate can be estimated as the product of the highest rate of recombination – 0.011 by the largest distance between the loci-1.32 Mbp, = 1.5*10^-2^.

The average of mutation rate in the HLA loci is 0.5*10^-5^ up to 2.5*10^-5^  per generation and per locus ([Turpeinen, Volin et al. 2009](#_ENREF_6)).

# References

Cullen, M., J. Noble, H. Erlich, K. Thorpe, S. Beck, W. Klitz, J. Trowsdale and M. Carrington (1997). "Characterization of recombination in the HLA class II region." American journal of human genetics **60**(2): 397.

Cullen, M., S. P. Perfetto, W. Klitz, G. Nelson and M. Carrington (2002). "High-resolution patterns of meiotic recombination across the human major histocompatibility complex." The American Journal of Human Genetics **71**(4): 759-776.

MHC_sequencing_consortium (1999). "Complete sequence and gene map of a human major histocompatibility complex. The MHC sequencing consortium." Nature **401**(6756): 921-923.

Turpeinen, H., L. Volin, L. Nikkinen, P. Ojala, A. Palotie, J. Saarela and J. Partanen (2009). "Genetic similarity of chromosome 6 between patients receiving hematopoietic stem cell transplantation and HLA matched sibling donors." Haematologica **94**(4): 528-535.

Yeager, M. and A. L. Hughes (1999). "Evolution of the mammalian MHC: natural selection, recombination, and convergent evolution." Immunological reviews **167**(1): 45-58.
