# Supplementary material for: HLA class I haplotype diversity is consistent with selection for frequent existing haplotypes
Source: PLoS Comput Biol. 2017 Aug 28;13(8):e1005693. doi: 10.1371/journal.pcbi.1005693 (PMC5590998; doi:10.1371/journal.pcbi.1005693)
Supplement: S1 Text — (DOCX) [file pcbi.1005693.s003.docx]

# S1 Text. Description of Simulations.

Four types of simulations were performed (beyond the standard neutral evolution): two for the neutral evolution, and two for positive frequency-dependent selection (FDS). The neutral simulations were performed for constant or growing populations, while FDS was simulated for a constant population. Simulations were performed over haplotypes composed of two alleles each. The alleles are characterized by a serial number. There is no explicit limit to the number of possible alleles or haplotypes in the simulation.

All simulations started with a neutral simulation of 1e7 individuals each with a single haplotype composed of two alleles, starting from a Ewens distribution of the alleles and random mixing of alleles. The simulations were then run for 1.e11 single event time steps, where each event represents the replacement of an existing haplotype by another. Specifically, these events are:

- Reproduction, where one haplotype replaces another haplotype in the population (we did not explicitly model genotypes).
- Mutation, where a new allele occurs in one of the haplotypes following reproduction. Specifically, following the reproduction event mentioned above, one of the alleles in the target haplotype is replaced by a new allele. We assume an infinite site model. Thus mutations can never reproduce an existing allele.
- Recombination, where two haplotypes are mixed. Specifically, two original haplotypes are chosen and the first allele from the first haplotype and second allele from the second haplotype are chosen to produce a new allele that can replace a randomly chosen other haplotype.

The simulations were performed with either a 1e-2 recombination and 1e-3 mutation rates, or 1e-1 recombination and 1e-5 mutation rates.

Following the initial period (the 1.e11 steps mentioned above), four different scenarios were tested:

1. **Sub-population Structure.** The population was divided into two equal sub-populations randomly. In each reproduction/recombination step, a random haplotype was chosen, and the target haplotype to be replaced was chosen from its own sub-population with a probability of alpha, or from the entire population (either within its own population or the other sub-population randomly) with a probability of (1-alpha) defined as the mixing fraction. In the case of recombination, the source haplotype for the recombination was chosen with probability alpha from the same sub-population as the target haplotype and with probability (1-alpha) from any of the sub-populations.
2. **Bottleneck.** The population was expanded to triple its original size. Given an initial population size of N, 3N positions were allowed for the target of reproduction events. If the target of the reproduction was empty, or a new haplotype was created (i.e. a new copy of an existing haplotype). Otherwise, an existing haplotype was replaced. The haplotype frequency distribution was sampled every 10 generations after the growth event. Each generation represents N reproduction events.
3. **Constant Population with FDS.** Each population was maintained at a constant size by offsetting each new individual with the death of an existing individual in the population. The death probability was not affected by the haplotype frequency, however haplotype frequency did influence generation of new individuals. Haplotypes that occurred between 1 and 100 times had their probability adjusted by a factor of between 1 and 10 (linearly interpolated), with a fixed 10x adjustment for more frequent haplotypes above the T1-count threshold. Mutation and recombination events were similar to the neutral model. T1 was varied from 100 to 1000.
4. **Viability based FDS simulation**. The same simulation was run where the death probability decreases linearly with the population size, and the division probability is constant.
