# Supplementary material for: HLA class I haplotype diversity is consistent with selection for frequent existing haplotypes
Source: PLoS Comput Biol. 2017 Aug 28;13(8):e1005693. doi: 10.1371/journal.pcbi.1005693 (PMC5590998; doi:10.1371/journal.pcbi.1005693)
Supplement: S2 Text — (DOCX) [file pcbi.1005693.s004.docx]

S2 Text. Simulation results.

In the following sections, we describe simulation results, and compare them to the observed D’ values and observed differences between observed and compare them to the observed results. Briefly, we first report the observed values, which are A) higher than expected F values in haplotypes and lower than expected F values in alleles, and B) decreasing and then increasing D’ values as a function of the haplotype frequencies. We then show that such patterns can be observed in simulations with selection, and that such patterns are not observed in standard simulations without selection, with either bottleneck, population sub-structure. This is obviously not an evidence that such behavior cannot be observed in any simulation. In section S5.4 we also show that these results are not a sample effect.

The current Supp. Mat section contains the following sub-sections.

**S2.1 Observed F and D’ values.**

**S2.2 Text. D’ estimated from population sub-structure and growing populations.**

**S2.3. Simulation of positive FDS.**

**S2.4 Figure. Effect of Sample Size on Fnd Values.**

**S2.5 Figure. Distribution of Fnd Values in Simulations.**

**S2.6 Figure - Simulation with viability based positive and negative FDS.**

**S2.7 Text - Details of Models studied.**

# S2.1 Observed F and D’ values.


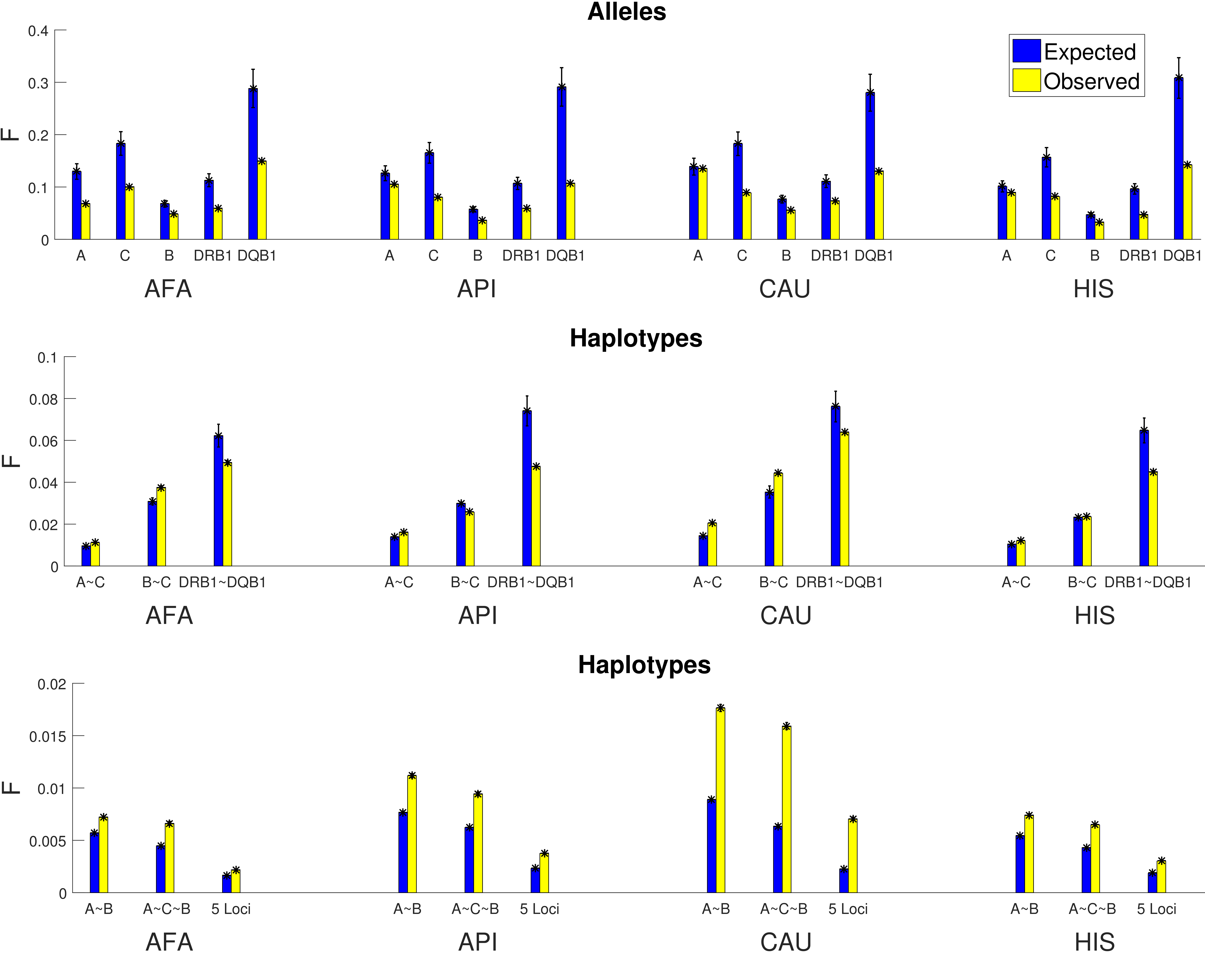


The figure above are the F values from the observed populations. We have computed the expected (blue bars) and observed (yellow bars) F values with error bars representing the standard deviation over samples. We present here the results for 4 large populations. The results for all populations are in Supp. Mat. S2. The different plots are different allele combinations or the full 5 locus haplotypes. One can clearly see that in the top figures the blue lines are significantly higher than the yellow lines (higher expected than observed homozygosity), and in the bottom plot, the inverse happens. The plot below is a repetition of figure 4 in the text.


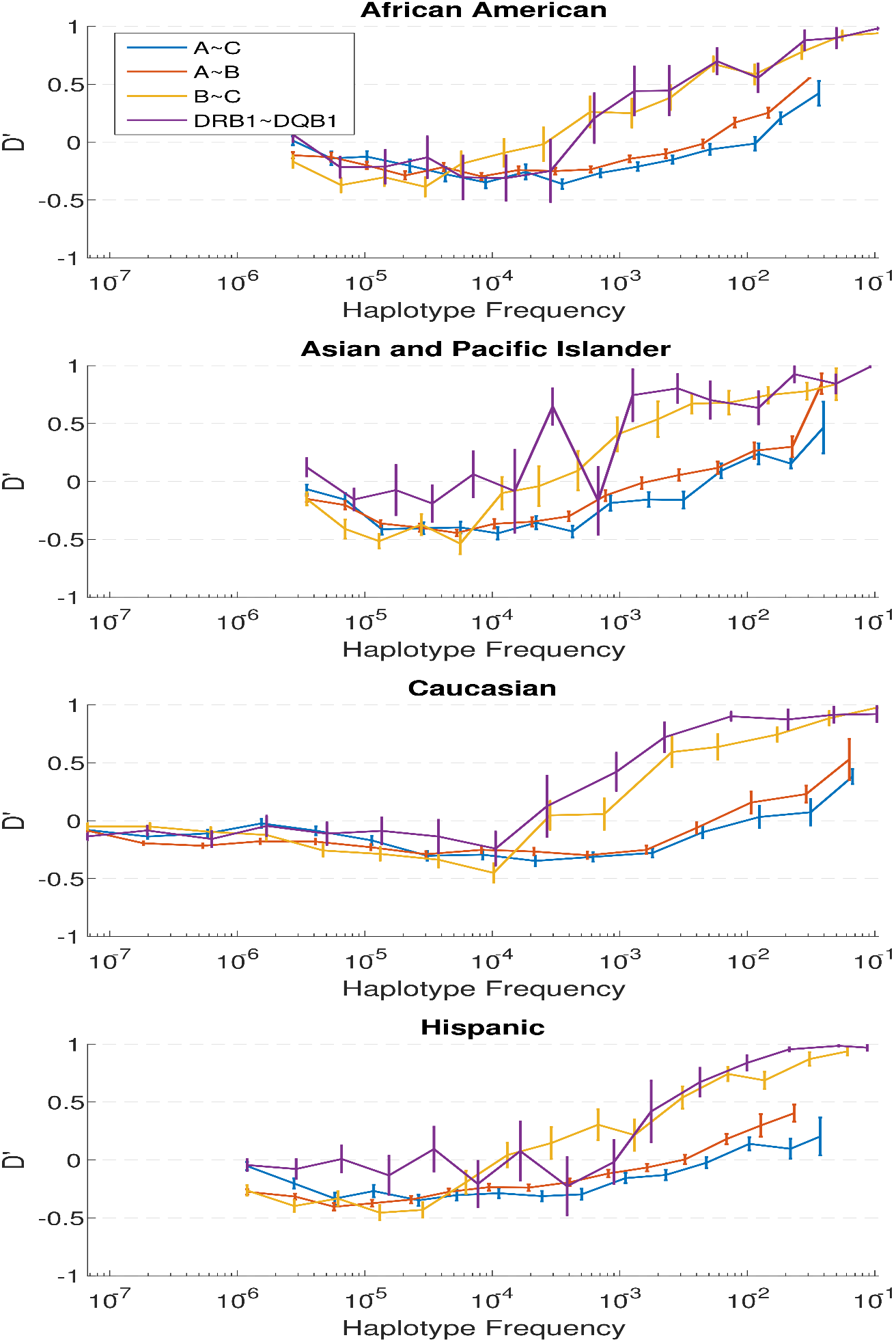


Figure 4 for the main text.

# S2.2 D’ estimated from population sub-structure and growing populations

Two measures of linkage disequilibrium (LD) were computed for each simulation. The first LD measure is the Lewontin Normalized D’ ([Lewontin 1964](#_ENREF_3)), the second is the correlation -$D/\sqrt{p_{i}(1-p_{i})p_{j}(1-p_{j})}$. We run two types of simulations each with two sets of mutations and recombination rates as described in S3. In all simulations, the LD started high and decreased to 0 as a function of the allele frequency product. These simulation results is in clear contrast with the results observed from our HLA haplotype frequency data where high LD has been maintained until today. The high normalized LD values for very low allele frequency products are easy to understand. Assume a recent recombination event of two rare alleles, the product of frequency for any pair of rare alleles would be extremely low, however any new haplotype would have a frequency of at least 1 divided by the population size. Therefore the frequency of the newly observed haplotype would be relatively much higher than what would be expected based on the rare allele frequencies.

The LD results are presented as a function of the allele frequency product and the fraction of mixing (or the time following the bottleneck). A large number of other simulations were performed with similar results. The upper plots represent a mutation rate of 1.e-4 and a recombination rate of 1.e-3. The lower plots represent a mutation rate of 1.e-5 and a recombination rate of 1.e-1.

# References

Lewontin, R. (1964). "The interaction of selection and linkage. I. General considerations; heterotic models." Genetics **49**(1): 49-67.


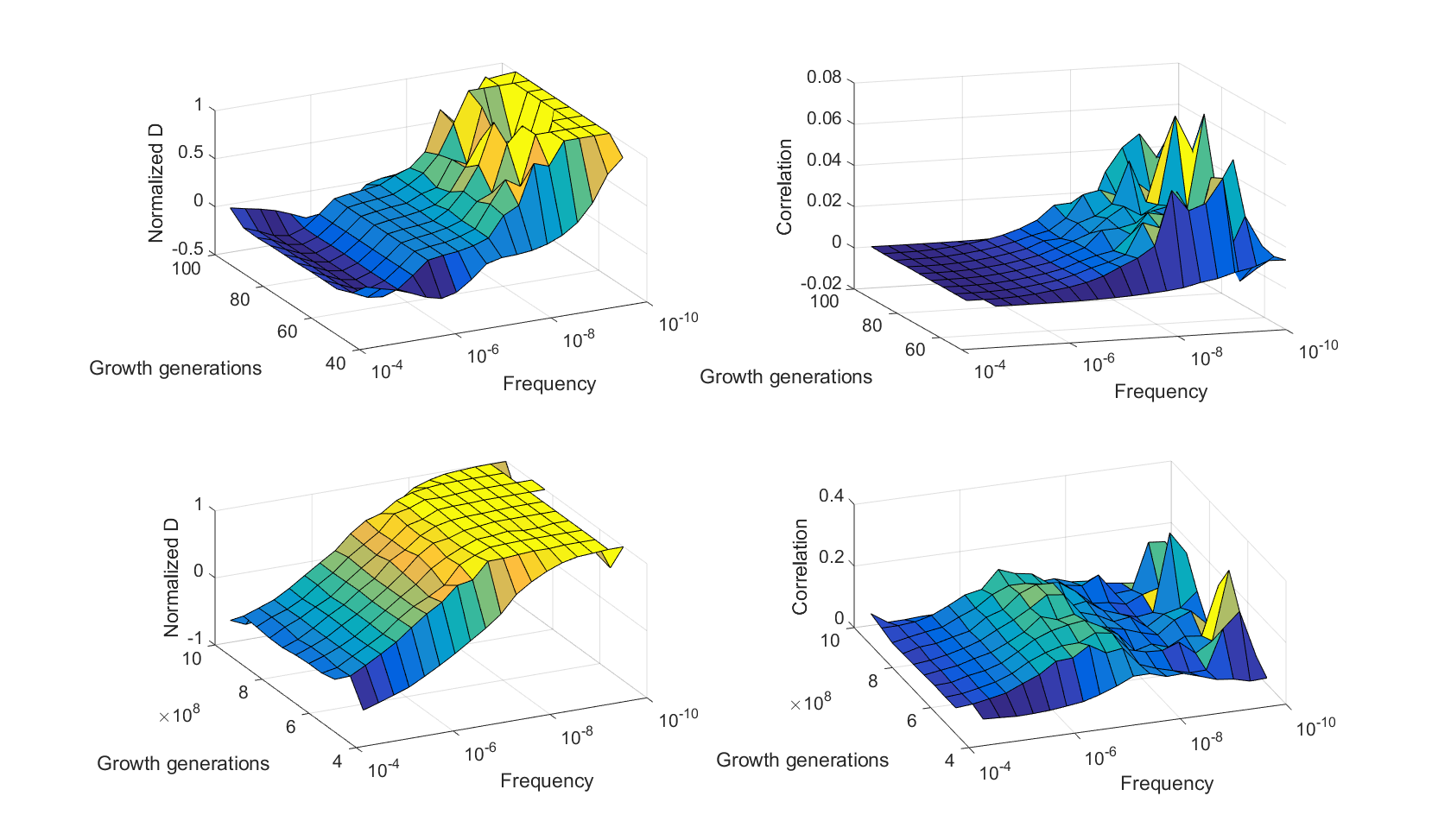

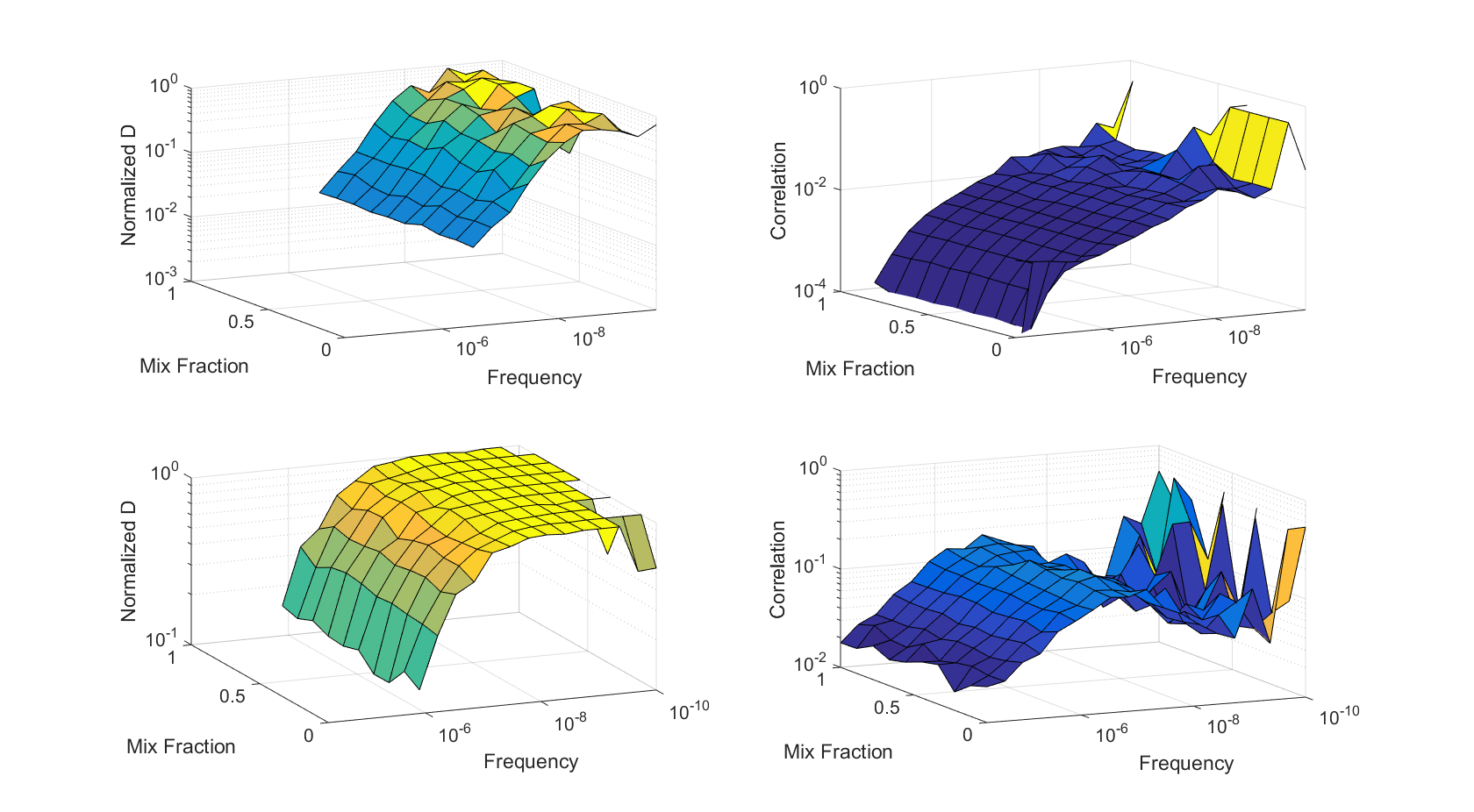


# S2.3 Figure. Simulation of positive FDS.

We simulated positive FDS with a uniform death probability and creation probability proportional to the frequency up to a level of 100 individuals and flat above that. We then computed the LD as the Lewontin normalized D’ value. The LD was computed every 100 time steps, where a single time step represents in average one creation event and one death event per organism. One can see that the LD increases with the allele frequency product in contrast with the neutral simulations. Note that this is just a proof of concept and we do not fit the actual observed distribution. Moreover, the observed distribution may be the result of purifying selection and not positive FDS.


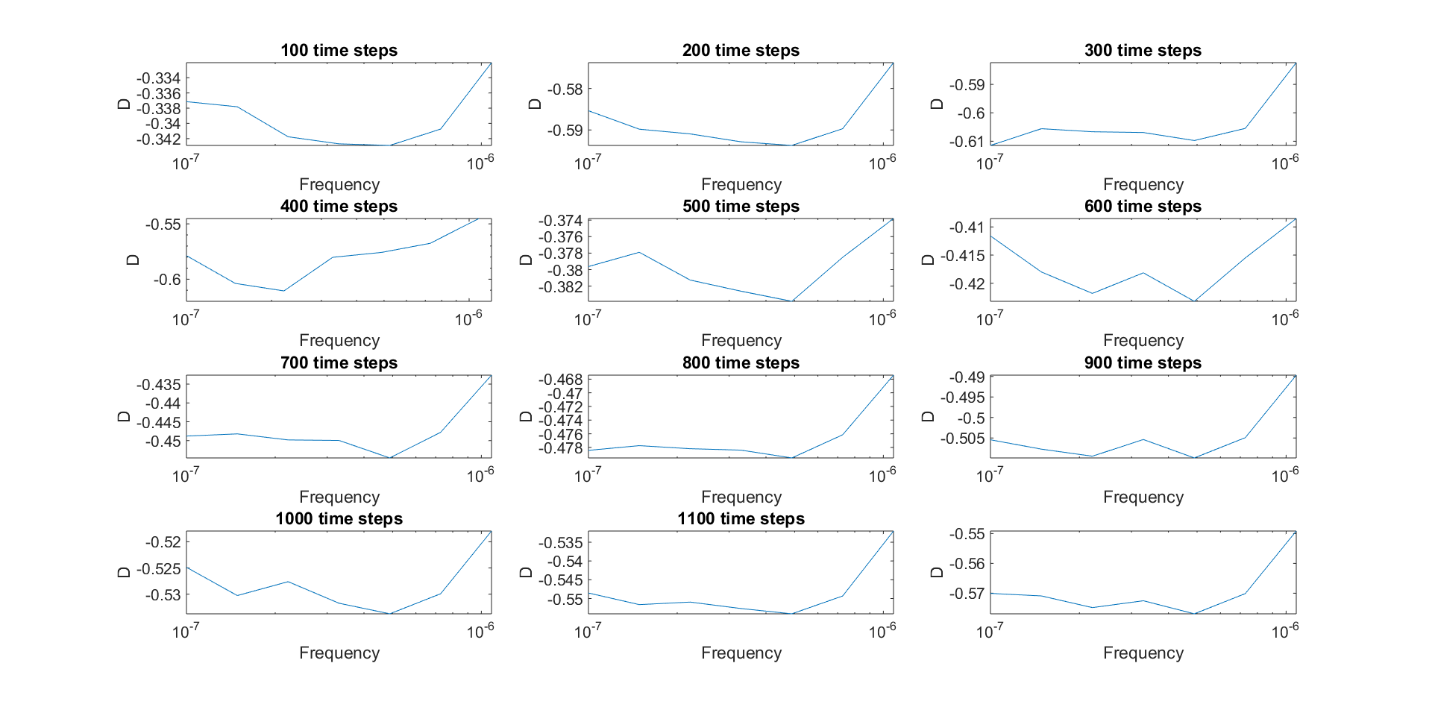


# S2.4 Figure. Effect of Sample Size on Fnd Values


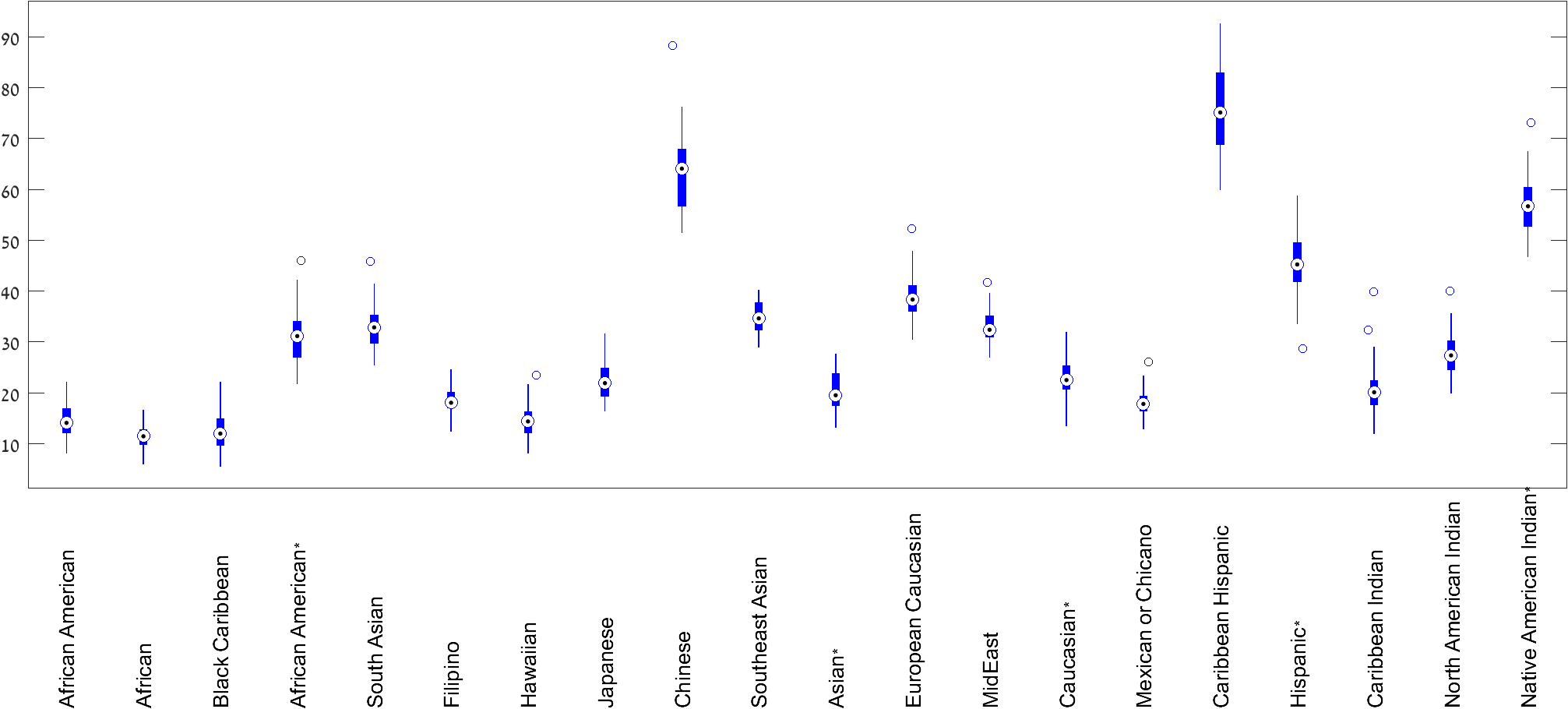


In order to apply Slatkin's method for the EW test, we randomly sampled a small number of subjects from each sub-population, and performed the test on the sample. In order to verify that the results are indeed representative, we repeated this process independently 100 times and report here the distribution of the Fnd values calculated. As can be observed, the samples do not differ by much. Note that even the largest samples are a few orders larger than 1,200. As such the sample with returns has a limited effect on the results.

Wide bars indicate the interquartile range, with the median indicated by a circled dot. Narrow bars extend up to 1.5 of the interquartile range.

# S2.5 Figure. Distribution of Fnd Values in Simulations

The expected and observed Fnd values of haplotypes were computed for the simulations described in S3 and S5. The recombination and mutation rates are similar to the realistic values computed in S4. The Fnd values are computed as the average over 25 samples each performed from an independent simulation.. The x-axes are similar to S5 either the mixing fraction or the time from the bottleneck. Specifically, in the upper plots, two simulations are shown with a population sub-structure and different mixing fractions, and in the lower plots two simulations are shown with different times from the bottleneck. The yellow plots are the observed Fnd values, and the blue plots are the expected Fnd values for a sample of 1,200 haplotypes. The left column represents a mutation rate of 1.e-4 and a recombination rate of 1.e-3. The right column represents a mutation rate of 1.e-5 and a recombination rate of 1.e-1.

One can clearly observe that while some minor, yet significant, deviations are observed, they never approach the scale of the deviations observed in the real HLA haplotype frequency distributions. None of the other population simulations we performed produced the high haplotype Fnd value differences (and thus high Fnd values). Moreover, none of the simulations performed produced opposite Fnd values in alleles and haplotypes.


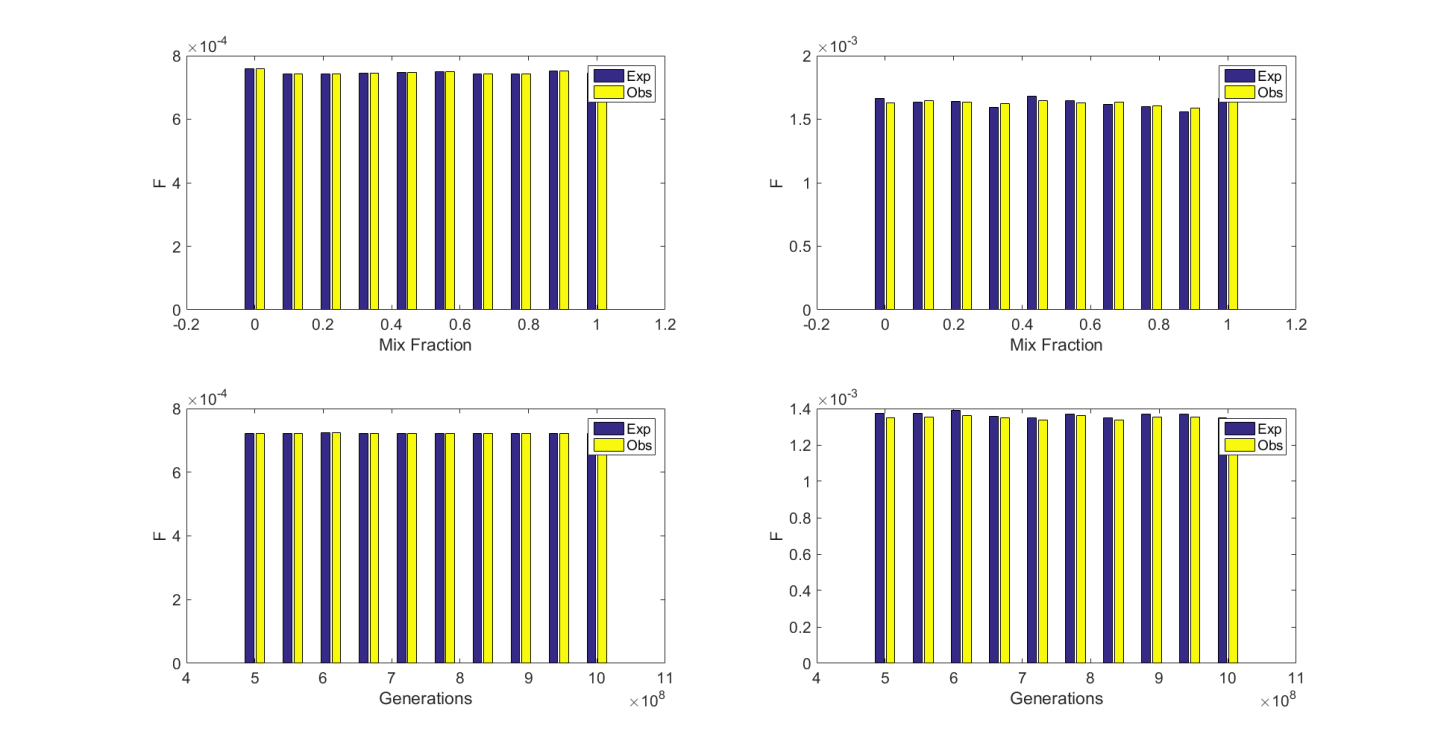


# S2.6 Figure - Simulation with viability based positive and negative FDS.

In order to test whether a combination of purifying and balancing selection on haplotypes and alleles can lead to the observed opposite deviation from neutral homozygosity, we simulated a viability bases selection model, where the probability of division, mutation and recombination are not affected by the allele or haplotype. However, the death rate is affected by the frequency of the alleles and haplotypes, with the following reactions:

- Constant birth rate.
- Constant mutation rate.
- Death rate of a person with haplotype H, and alleles A1 and A2, defined by :

$f(H)=\frac{\prod_{i=1,2} g(A_{i})}{g(H)}$, where

The function $g(x)$ represents the selection. If $g(x)=const$, there is no selection, and the model is neutral, and when $\theta>0$, then alleles induce a negative FDS, and haplotypes induce a positive FDS.

We here assumed different scales of selection $x_{\max}=10,\theta=1,x_{0}=0$for the alleles, and$x_{\max}=100,\theta_{H}=1,x_{0}=0$ for the haplotypes. These are obviously arbitrary values, and are only used to show the possibility of the proposed mechanism. We do not argue here that these parameters represent any real mechanism, only that such opposite results are possible.

When no selection is assumed on haplotypes$\theta_{H}=0$, but selection is assumed on alleles, then alleles homozygosity is lower than expected, and haplotype homozygosity is as the neutral one. (upper plot below). When both selection types are simulated, alleles and haplotypes show opposite deviations from the homozygosity expected from a neutral model (lower plot below).


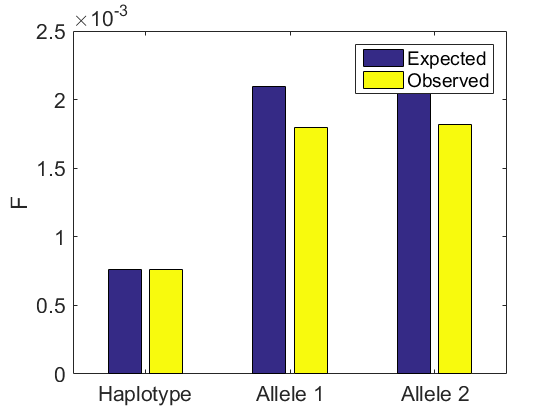

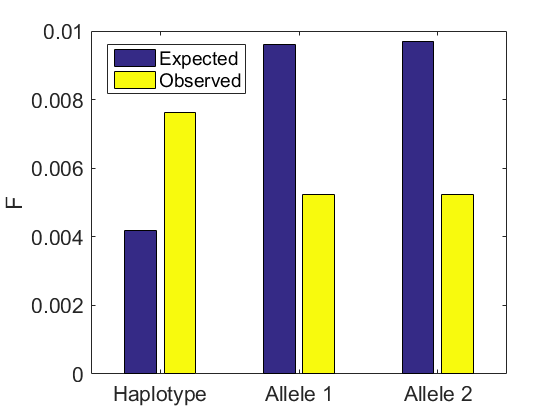


# S2.7 Text - Details of Models studied

The expected frequency distributions of the three theoretical models were compared. All theoretical models were developed for allele frequencies, and have explicit solutions for the allele frequency distribution.

1. The constant population neutral drift model. In this model, the population is constant, and each division event is paralleled by a death event. Each division event leads to the reproduction of the same organism with a probability of$(1-\mu)$ and to the creation of a novel mutant with a probability of$\mu$. This is formally a neutral Moran model ([Moran 1958](#_ENREF_5)).
2. A growing population neutral model. In this model, the population grows exponentially with no death included. Each division event leads to the reproduction of the same organism with probability $(1-\mu)$ and to the creation of a novel mutant with a probability of$\mu$. Since, no death is modeled, each division event leads to a population growth. This is formally a Yule-Willis model ([Yule 1925](#_ENREF_8)).
3. The last model is described at the level of populations and not at the level of individuals. In this model each sub-population grows with a rate of $\lambda(x+a)$ and shrinks with a rate of $\eta(x+b)$, where x is the sub-population size. The left boundary condition on x is determined by the mutation rate. Note that ratio of growth to death rate of new haplotypes is $\lambda a/\eta b$, while for large haplotypes, this ratio approaches $\lambda/\eta$. Thus positive values of $\delta=b-a$imply an advantage for large population over small populations.

# References

Moran, P. A. P. (1958). Random processes in genetics. Proceedings of the Cambridge Philosophical Society.

Yule, G. (1925). "A mathematical theory of evolution, based on the conclusions of Dr. JC Willis, FRS." Philosophical Transactions of the Royal Society of … **213**: 21-87.
