# Supplementary material for: HLA class I haplotype diversity is consistent with selection for frequent existing haplotypes
Source: PLoS Comput Biol. 2017 Aug 28;13(8):e1005693. doi: 10.1371/journal.pcbi.1005693 (PMC5590998; doi:10.1371/journal.pcbi.1005693)
Supplement: S1 Appendix — (DOCX) [file pcbi.1005693.s005.docx]

# S1 Appendix. Ewens-Watterson Test Results for Single Alleles and haplotypes

Average *Fnd* computed using the Ewens-Watterson test performed on subsamples of 1,200 individuals from the 23 race groups in the US population. We show for each locus tested and each group the p-value of the test (percentile of the sample homozygosity value) and the *Fnd.*

**Table A. *Fnd* values for five HLA alleles using Arlequin**

| Race Group | HLA-A | HLA-C | HLA-B | HLA-DRB1 | HLA-DQB1 | HLA-A~C | HLA-A~B | HLA-B~C | HLA-DRB1~DQB1 | HLA-A~C~B | 5 Loci |
| --- | --- | --- | --- | --- | --- | --- | --- | --- | --- | --- | --- |
| African American | -1.48 | -1.23 | -0.99 | -1.34 | -1.24 | 1.05 | 5.22 | 1.59 | -1.05 | 8.69 | 17.48 |
| African | -1.41 | -1.03 | -0.84 | -1.36 | -1.22 | 2.00 | 5.02 | 1.78 | -0.78 | 6.15 | 12.13 |
| Black Caribbean | -1.42 | -1.05 | -0.64 | -1.30 | -1.07 | 2.31 | 4.80 | 3.15 | -0.21 | 8.73 | 15.78 |
| **African American*** | -1.42 | -1.21 | -1.06 | -1.30 | -1.23 | 2.10 | 3.29 | 1.25 | -0.95 | 10.21 | 20.13 |
| South Asian | -0.92 | -1.26 | -1.18 | -0.64 | -1.46 | 2.48 | 6.52 | -0.04 | -0.65 | 6.40 | 33.52 |
| Filipino | -0.15 | -0.73 | -0.55 | 1.51 | -1.01 | 3.20 | 5.74 | 1.96 | 1.43 | 8.63 | 27.35 |
| Hawaiian or other Pacific Islander | 0.21 | -1.20 | -0.44 | -1.05 | -1.45 | 1.63 | 6.27 | 1.08 | -0.96 | 9.61 | 23.83 |
| Japanese | 0.19 | -1.09 | -1.05 | -1.17 | -1.55 | 0.28 | 1.64 | 0.20 | -0.93 | 4.03 | 32.32 |
| Korean | -0.74 | -1.48 | -1.44 | -1.55 | -1.71 | -0.26 | 4.10 | -1.01 | -1.42 | 4.94 | 30.40 |
| Chinese | -0.27 | -0.94 | -0.11 | -1.21 | -1.48 | 3.39 | 9.52 | 1.93 | -0.77 | 14.18 | 37.34 |
| Other Southeast Asian | -0.81 | -1.41 | -1.35 | -1.03 | -1.41 | 1.96 | 5.08 | -0.20 | -0.62 | 8.62 | 26.32 |
| Vietnamese | -0.70 | -1.29 | -0.68 | -0.03 | -0.93 | 5.30 | 13.01 | 1.98 | 1.26 | 22.31 | 49.31 |
| **Asian and Pacific Islander*** | -0.28 | -1.40 | -1.33 | -1.28 | -1.67 | 1.65 | 3.98 | -0.09 | -1.04 | 7.36 | 16.97 |
| European Caucasian | -0.26 | -1.19 | -1.03 | -1.02 | -1.29 | 4.89 | 13.04 | 1.02 | -0.58 | 19.63 | 63.66 |
| MidEast/No. Coast of Africa | -0.22 | -1.28 | -1.45 | -1.37 | -1.21 | 3.21 | 4.91 | -0.49 | -0.86 | 6.53 | 19.43 |
| **Caucasian*** | 0.13 | -1.25 | -0.81 | -0.68 | -1.38 | 3.78 | 11.17 | 0.78 | -0.62 | 15.91 | 58.26 |
| Mexican or Chicano | -0.70 | -1.26 | -1.14 | -1.34 | -1.19 | 2.43 | 3.29 | -0.42 | -0.92 | 6.46 | 15.23 |
| South/Cntrl Amer. Hisp. | -0.05 | -1.20 | -1.04 | -1.55 | -1.37 | 1.51 | 7.11 | -0.04 | -1.02 | 8.83 | 25.25 |
| Caribbean Hispanic | -1.13 | -1.34 | -1.36 | -1.52 | -1.31 | 0.43 | 3.74 | -1.02 | -1.17 | 7.98 | 24.81 |
| **Hispanic*** | -0.61 | -1.23 | -1.10 | -1.49 | -1.30 | 2.63 | 5.49 | 0.18 | -1.27 | 7.55 | 19.18 |
| Caribbean Indian | -1.04 | -1.48 | -1.11 | -1.19 | -1.46 | -0.60 | 4.95 | -1.04 | -0.87 | 5.89 | 18.38 |
| North American Indian | -0.27 | -1.35 | -0.87 | -1.38 | -1.32 | 2.17 | 5.57 | 0.73 | -1.29 | 10.33 | 28.19 |
| **Native American Indian*** | -0.10 | -1.37 | -0.80 | -1.50 | -1.38 | 3.63 | 6.29 | 1.00 | -0.90 | 10.11 | 38.68 |

*Broad race groups, composed of the detailed race groups above it in the table

**Table B. Uncorrected p-values of Slatkin's exact test for five HLA alleles using Arlequin –** Bolded p-values remain significant at the 0.05 level following a multiple measurement correction.

| Race Group | HLA A | HLA-C | HLA-B | HLA-DRB1 | HLA-DQB1 | HLA-A~C | HLA-A~B | HLA-B~C | HLA-DRB1~DQB1 | HLA-A~C~B | 5 Loci |
| --- | --- | --- | --- | --- | --- | --- | --- | --- | --- | --- | --- |
| African American | 8.80E-03 | **1.96E-02** | 1.57E-01 | 1.07E-01 | **2.26E-02** | 0.8414 | **1** | **0.9777** | 0.3166 | **1** | **1** |
| African | 5.06E-02 | 7.24E-02 | 6.10E-01 | 5.57E-02 | 3.99E-02 | **0.992** | **1** | **0.997** | 0.1813 | **1** | **1** |
| Black Caribbean | 5.82E-02 | 1.15E-01 | 6.90E-01 | 1.08E-01 | 9.57E-02 | **0.9884** | **1** | **1** | 0.8977 | **1** | **1** |
| **African American*** | 5.50E-03 | **8.80E-03** | 1.81E-01 | 7.62E-02 | 4.75E-02 | **0.9866** | **0.9991** | **0.9903** | 0.2379 | **1** | **1** |
| South Asian | 2.26E-01 | 2.68E-02 | 2.57E-01 | 6.11E-01 | **3.80E-03** | **0.9831** | **1** | 0.9742 | 0.8496 | **1** | **1** |
| Filipino | 7.70E-01 | **1.42E-02** | 4.73E-01 | 8.65E-01 | **1.72E-02** | **1** | **1** | **0.9958** | 0.8142 | **1** | **1** |
| Hawaiian or other Pacific Islander | 9.26E-01 | 5.51E-02 | 1.58E-01 | 2.81E-01 | **1.70E-03** | **0.9993** | **1** | 0.9476 | 0.1973 | **1** | **1** |
| Japanese | 8.83E-01 | 2.75E-01 | 4.55E-01 | 6.57E-02 | **5.70E-03** | 0.9596 | **0.9836** | **0.9979** | 0.0878 | **0.9999** | **1** |
| Korean | 2.01E-01 | **2.00E-03** | 7.98E-02 | **1.34E-02** | **0.00E+00** | 0.9116 | **1** | 0.4876 | 0.0312 | **1** | **1** |
| Chinese | 6.68E-01 | 1.64E-01 | 4.65E-01 | 8.09E-02 | **2.00E-03** | **1** | **1** | **0.9884** | 0.4083 | **1** | **1** |
| Other Southeast Asian | 1.24E-01 | 4.64E-02 | 5.46E-02 | 4.56E-01 | **7.60E-03** | 0.9632 | **0.9998** | 0.9313 | 0.7135 | **1** | **1** |
| Vietnamese | 1.02E-01 | 3.05E-02 | 1.64E-01 | 4.82E-01 | 8.57E-02 | **1** | **1** | **0.9876** | 0.9007 | **1** | **1** |
| **Asian and Pacific Islander*** | 5.54E-01 | **4.20E-03** | 5.15E-02 | 2.61E-01 | **0.00E+00** | **0.9983** | **1** | 0.974 | 0.6584 | **1** | **1** |
| European Caucasian | 2.18E-01 | 1.51E-01 | 6.62E-02 | 1.08E-01 | **1.41E-02** | **0.9995** | **1** | **0.9807** | 0.8409 | **1** | **1** |
| MidEast/No. Coast of Africa | 5.55E-01 | **7.90E-03** | 5.46E-02 | **2.30E-03** | **3.50E-03** | **0.9996** | **1** | 0.8871 | 0.265 | **1** | **1** |
| **Caucasian*** | 7.36E-01 | 1.64E-01 | 4.68E-01 | 6.38E-01 | **1.47E-02** | **0.9909** | **1** | 0.9455 | 0.691 | **1** | **1** |
| Mexican or Chicano | 1.22E-01 | 3.16E-02 | 2.58E-01 | **1.87E-02** | 9.56E-02 | **0.9891** | **0.9997** | 0.769 | 0.4376 | **1** | **1** |
| South/Cntrl Amer. Hisp. | 6.92E-01 | 5.93E-02 | 1.47E-01 | **2.30E-03** | **6.20E-03** | **0.9754** | **1** | 0.9168 | 0.3188 | **1** | **1** |
| Caribbean Hispanic | 4.21E-02 | **4.60E-03** | 2.18E-02 | **0.00E+00** | 3.55E-02 | 0.4935 | **0.9875** | 0.2953 | 0.0109 | **1** | **1** |
| **Hispanic*** | 1.69E-01 | 2.90E-02 | 4.21E-01 | **9.00E-04** | 3.71E-02 | **0.9972** | **1** | **0.9841** | 0.0468 | **1** | **1** |
| Caribbean Indian | 1.49E-01 | **7.00E-04** | 2.06E-01 | **7.50E-03** | **2.80E-03** | 0.0747 | **1** | 0.4262 | 0.1615 | **1** | **1** |
| North American Indian | 4.04E-01 | **1.06E-02** | 3.32E-01 | **3.20E-03** | 4.17E-02 | **0.9952** | **1** | 0.9722 | 0.0346 | **1** | **1** |
| **Native American Indian*** | 6.15E-01 | **6.30E-03** | 3.84E-01 | **7.00E-04** | **4.60E-03** | **0.9984** | **1** | 0.9651 | 0.3195 | **1** | **1** |

| **Table C-Observed homozygosity in samples of size 1200 (N alleles/haplotypes) from HLA allele and haplotype frequencies as computed from Pypop.** | | | | | | | | | | | |
| --- | --- | --- | --- | --- | --- | --- | --- | --- | --- | --- | --- |
| Race group \ Locus | HLA-A | HLA-C | HLA-B | HLA-DRB1 | HLA-DQB1 | HLA-A~C | HLA-A~B | HLA-B~C | HLA-DRB1~DQB1 | HLA-A~C~B | 5 Locus |
| African American | 0.0663 | 0.0959 | 0.0503 | 0.0595 | 0.1497 | 0.0108 | 0.0076 | 0.0381 | 0.0539 | 0.0064 | 0.0022 |
| African | 0.0646 | 0.1094 | 0.0445 | 0.0654 | 0.1453 | 0.0104 | 0.0065 | 0.0385 | 0.0525 | 0.0059 | 0.0021 |
| Black Caribbean | 0.0637 | 0.1028 | 0.0546 | 0.0642 | 0.146 | 0.0112 | 0.0072 | 0.0382 | 0.0577 | 0.0071 | 0.0021 |
| **African American*** | 0.0659 | 0.0978 | 0.0491 | 0.0609 | 0.1486 | 0.0106 | 0.0072 | 0.039 | 0.049 | 0.007 | 0.0022 |
| South Asian | 0.0894 | 0.0937 | 0.0499 | 0.0794 | 0.1327 | 0.0179 | 0.011 | 0.0422 | 0.0654 | 0.0103 | 0.0049 |
| Filipino | 0.1463 | 0.1578 | 0.0544 | 0.1717 | 0.1736 | 0.0349 | 0.0218 | 0.047 | 0.1211 | 0.0176 | 0.0111 |
| Hawaiian or other Pacific Islander | 0.1277 | 0.0981 | 0.0532 | 0.065 | 0.1325 | 0.0256 | 0.0166 | 0.0378 | 0.0594 | 0.0146 | 0.0081 |
| Japanese | 0.1823 | 0.1169 | 0.065 | 0.0788 | 0.1099 | 0.0306 | 0.0262 | 0.0534 | 0.0793 | 0.023 | 0.0126 |
| Korean | 0.1281 | 0.0873 | 0.0481 | 0.0604 | 0.0904 | 0.0236 | 0.0184 | 0.0369 | 0.0536 | 0.0157 | 0.0088 |
| Chinese | 0.1402 | 0.1171 | 0.0666 | 0.0763 | 0.12 | 0.0335 | 0.0263 | 0.0533 | 0.0678 | 0.0268 | 0.0084 |
| Other Southeast Asian | 0.0919 | 0.0744 | 0.0367 | 0.0658 | 0.1235 | 0.0154 | 0.0112 | 0.03 | 0.056 | 0.0099 | 0.0042 |
| Vietnamese | 0.1217 | 0.1006 | 0.0669 | 0.1201 | 0.168 | 0.0322 | 0.0231 | 0.0551 | 0.1124 | 0.0237 | 0.011 |
| **Asian and Pacific Islander*** | 0.103 | 0.0798 | 0.0365 | 0.0596 | 0.1031 | 0.0165 | 0.011 | 0.0264 | 0.0471 | 0.0106 | 0.0038 |
| European Caucasian | 0.1456 | 0.0896 | 0.0655 | 0.0797 | 0.1496 | 0.0231 | 0.0225 | 0.0485 | 0.0716 | 0.0192 | 0.0076 |
| MidEast/No. Coast of Africa | 0.096 | 0.0853 | 0.0391 | 0.07 | 0.1408 | 0.0142 | 0.0095 | 0.0279 | 0.0574 | 0.0079 | 0.0029 |
| **Caucasian*** | 0.1384 | 0.0879 | 0.0575 | 0.0733 | 0.1285 | 0.0227 | 0.0188 | 0.0444 | 0.0653 | 0.017 | 0.007 |
| Mexican or Chicano | 0.0975 | 0.0875 | 0.0344 | 0.0525 | 0.1518 | 0.0134 | 0.0094 | 0.0267 | 0.05 | 0.0077 | 0.0032 |
| South/Cntrl Amer. Hisp. | 0.0913 | 0.0844 | 0.033 | 0.046 | 0.1416 | 0.0118 | 0.0069 | 0.0221 | 0.0403 | 0.0068 | 0.0027 |
| Caribbean Hispanic | 0.0653 | 0.0775 | 0.0341 | 0.0568 | 0.1363 | 0.0104 | 0.0088 | 0.0219 | 0.0493 | 0.0075 | 0.0046 |
| **Hispanic*** | 0.0854 | 0.0817 | 0.0334 | 0.05 | 0.1407 | 0.0117 | 0.0074 | 0.0234 | 0.0474 | 0.0064 | 0.0028 |
| Caribbean Indian | 0.0614 | 0.0858 | 0.037 | 0.0575 | 0.1322 | 0.0098 | 0.0079 | 0.0198 | 0.0449 | 0.0073 | 0.0041 |
| North American Indian | 0.1354 | 0.0884 | 0.0564 | 0.0615 | 0.1381 | 0.0213 | 0.0158 | 0.0484 | 0.0516 | 0.0141 | 0.0067 |
| **Native American Indian*** | 0.1215 | 0.09 | 0.0521 | 0.0621 | 0.1408 | 0.0199 | 0.0152 | 0.0435 | 0.0526 | 0.0137 | 0.0064 |

**Table D - Expected homozygosity in samples of the same size (n=1200) and number of unique alleles as in table C**

| Race group \ Locus | HLA-A | HLA-C | HLA-B | HLA-DRB1 | HLA-DQB1 | HLA-A~C | HLA-A~B | HLA-B~C | HLA-DRB1~DQB1 | HLA-A~C~B | 5 Locus |
| --- | --- | --- | --- | --- | --- | --- | --- | --- | --- | --- | --- |
| African American | 0.1631 | 0.1754 | 0.0704 | 0.1124 | 0.2767 | 0.0098 | 0.0052 | 0.03 | 0.0629 | 0.0044 | 0.0016 |
| African | 0.1219 | 0.1631 | 0.0748 | 0.1124 | 0.2925 | 0.0096 | 0.0051 | 0.03 | 0.0595 | 0.0042 | 0.0015 |
| Black Caribbean | 0.142 | 0.1754 | 0.0664 | 0.1182 | 0.2925 | 0.009 | 0.0055 | 0.0268 | 0.0664 | 0.0045 | 0.0015 |
| **African American*** | 0.1293 | 0.198 | 0.0618 | 0.1152 | 0.3097 | 0.0095 | 0.0057 | 0.0364 | 0.0664 | 0.0047 | 0.0017 |
| South Asian | 0.1518 | 0.1754 | 0.0762 | 0.1152 | 0.2925 | 0.0134 | 0.0084 | 0.0431 | 0.0798 | 0.0068 | 0.0023 |
| Filipino | 0.1378 | 0.198 | 0.0664 | 0.1182 | 0.2767 | 0.0204 | 0.0116 | 0.0331 | 0.0813 | 0.0088 | 0.0039 |
| Hawaiian or other Pacific Islander | 0.1249 | 0.1827 | 0.0556 | 0.1152 | 0.2925 | 0.0178 | 0.0106 | 0.0307 | 0.0813 | 0.008 | 0.0037 |
| Japanese | 0.1827 | 0.198 | 0.1092 | 0.1378 | 0.3097 | 0.0279 | 0.0211 | 0.0556 | 0.1219 | 0.0137 | 0.0041 |
| Korean | 0.1901 | 0.1901 | 0.0934 | 0.1249 | 0.3097 | 0.0234 | 0.0142 | 0.0498 | 0.101 | 0.0102 | 0.0035 |
| Chinese | 0.142 | 0.1827 | 0.0652 | 0.1293 | 0.2925 | 0.0204 | 0.0116 | 0.036 | 0.0958 | 0.0087 | 0.0029 |
| Other Southeast Asian | 0.1249 | 0.1754 | 0.0618 | 0.1062 | 0.262 | 0.0122 | 0.0074 | 0.0282 | 0.0704 | 0.006 | 0.0022 |
| Vietnamese | 0.1572 | 0.1754 | 0.0798 | 0.1249 | 0.3097 | 0.0169 | 0.0094 | 0.0393 | 0.0868 | 0.0081 | 0.0036 |
| **Asian and Pacific Islander*** | 0.1219 | 0.1691 | 0.0576 | 0.1032 | 0.2925 | 0.013 | 0.0073 | 0.0303 | 0.0748 | 0.0066 | 0.0025 |
| European Caucasian | 0.1631 | 0.1754 | 0.0748 | 0.1182 | 0.3294 | 0.016 | 0.0094 | 0.0415 | 0.1032 | 0.0073 | 0.0024 |
| MidEast/No. Coast of Africa | 0.1293 | 0.1754 | 0.0607 | 0.1092 | 0.2925 | 0.0114 | 0.0065 | 0.0318 | 0.0748 | 0.005 | 0.0018 |
| **Caucasian*** | 0.1378 | 0.1901 | 0.069 | 0.1182 | 0.3097 | 0.015 | 0.0092 | 0.0352 | 0.069 | 0.0073 | 0.0022 |
| Mexican or Chicano | 0.1219 | 0.1572 | 0.0586 | 0.0982 | 0.3097 | 0.0105 | 0.0062 | 0.0313 | 0.0732 | 0.0049 | 0.0023 |
| South/Cntrl Amer. Hisp. | 0.1032 | 0.1631 | 0.0437 | 0.101 | 0.3097 | 0.0099 | 0.005 | 0.0199 | 0.0629 | 0.0042 | 0.0017 |
| Caribbean Hispanic | 0.1032 | 0.1827 | 0.0476 | 0.101 | 0.3097 | 0.0097 | 0.0058 | 0.0276 | 0.0732 | 0.0045 | 0.0021 |
| **Hispanic*** | 0.1092 | 0.1631 | 0.045 | 0.1032 | 0.3294 | 0.0108 | 0.0055 | 0.026 | 0.0607 | 0.0046 | 0.0018 |
| Caribbean Indian | 0.1032 | 0.1691 | 0.0515 | 0.1032 | 0.3097 | 0.0103 | 0.0063 | 0.0224 | 0.0629 | 0.0043 | 0.0024 |
| North American Indian | 0.1572 | 0.198 | 0.0748 | 0.0982 | 0.3294 | 0.0145 | 0.0086 | 0.0415 | 0.0813 | 0.0078 | 0.0027 |
| **Native American Indian*** | 0.1378 | 0.1691 | 0.0732 | 0.1124 | 0.2925 | 0.0138 | 0.0093 | 0.0378 | 0.0748 | 0.0067 | 0.0028 |

**Table E - Number of unique alleles (k) in the samples of table above**

| Race group \ Locus | HLA-A | HLA-C | HLA-B | HLA-DRB1 | HLA-DQB1 | HLA-A~C | HLA-A~B | HLA-B~C | HLA-DRB1~DQB1 | HLA-A~C~B | 5 Locus |
| --- | --- | --- | --- | --- | --- | --- | --- | --- | --- | --- | --- |
| African American | 28 | 26 | 60 | 40 | 16 | 272 | 418 | 120 | 66 | 465 | 851 |
| African | 37 | 28 | 57 | 40 | 15 | 276 | 422 | 120 | 69 | 481 | 870 |
| Black Caribbean | 32 | 26 | 63 | 38 | 15 | 290 | 404 | 131 | 63 | 462 | 866 |
| **African American*** | 35 | 23 | 67 | 39 | 14 | 278 | 393 | 103 | 63 | 444 | 820 |
| South Asian | 30 | 26 | 56 | 39 | 15 | 219 | 304 | 90 | 54 | 352 | 696 |
| Filipino | 33 | 23 | 63 | 38 | 16 | 161 | 242 | 111 | 53 | 294 | 505 |
| Hawaiian or other Pacific Islander | 36 | 25 | 73 | 39 | 15 | 178 | 259 | 118 | 53 | 314 | 516 |
| Japanese | 25 | 23 | 41 | 33 | 14 | 127 | 157 | 73 | 37 | 216 | 484 |
| Korean | 24 | 24 | 47 | 36 | 14 | 145 | 210 | 80 | 44 | 265 | 533 |
| Chinese | 32 | 25 | 64 | 35 | 15 | 161 | 242 | 104 | 46 | 296 | 604 |
| Other Southeast Asian | 36 | 26 | 67 | 42 | 17 | 234 | 332 | 126 | 60 | 382 | 717 |
| Vietnamese | 29 | 26 | 54 | 36 | 14 | 185 | 281 | 97 | 50 | 310 | 529 |
| **Asian and Pacific Islander*** | 37 | 27 | 71 | 43 | 15 | 224 | 335 | 119 | 57 | 356 | 662 |
| European Caucasian | 28 | 26 | 57 | 38 | 13 | 192 | 280 | 93 | 43 | 333 | 672 |
| MidEast/No. Coast of Africa | 35 | 26 | 68 | 41 | 15 | 246 | 362 | 115 | 57 | 430 | 808 |
| **Caucasian*** | 33 | 24 | 61 | 38 | 14 | 202 | 286 | 106 | 61 | 333 | 719 |
| Mexican or Chicano | 37 | 29 | 70 | 45 | 14 | 261 | 373 | 116 | 58 | 437 | 698 |
| South/Cntrl Amer. Hisp. | 43 | 28 | 89 | 44 | 14 | 271 | 431 | 164 | 66 | 476 | 830 |
| Caribbean Hispanic | 43 | 25 | 83 | 44 | 14 | 274 | 391 | 128 | 58 | 459 | 720 |
| **Hispanic*** | 41 | 28 | 87 | 43 | 13 | 255 | 401 | 134 | 68 | 455 | 806 |
| Caribbean Indian | 43 | 27 | 78 | 43 | 14 | 264 | 370 | 150 | 66 | 472 | 671 |
| North American Indian | 29 | 23 | 57 | 45 | 13 | 207 | 298 | 93 | 53 | 319 | 626 |
| **Native American Indian*** | 33 | 27 | 58 | 40 | 15 | 214 | 284 | 100 | 57 | 354 | 612 |

**Table F - Uncorrected p values of the Ewens Watterson test on the samples of table above (PyPop results)**

|  | | | | | | | | |  |  |  |
| --- | --- | --- | --- | --- | --- | --- | --- | --- | --- | --- | --- |
| Race group \ Locus | HLA-A | HLA-C | HLA-B | HLA-DRB1 | HLA-DQB1 | HLA-A~C | HLA-A~B | HLA-B~C | HLA-DRB1~DQB1 | HLA-A~C~B | 5 Locus |
| African American | **0.0004** | 0.0257 | 0.0985 | **0.0054** | 0.044 | 0.8264 | **0.9999** | 0.9123 | 0.3374 | **1** | **1** |
| African | **0.0056** | 0.1421 | 0.0096 | **0.0229** | **0.0215** | 0.7969 | **0.9959** | 0.9182 | 0.3789 | **1** | **1** |
| Black Caribbean | **0.0008** | 0.053 | 0.2711 | **0.0092** | **0.0224** | 0.9743 | **0.9981** | 0.9683 | 0.3656 | **1** | **1** |
| **African American*** | **0.0043** | **0.0109** | 0.2138 | **0.0049** | **0.013** | 0.8664 | **0.9932** | 0.7114 | 0.1232 | **1** | **1** |
| South Asian | 0.0448 | **0.021** | 0.0411 | 0.1238 | **0.0091** | 0.9791 | **0.9921** | 0.5478 | 0.3011 | **0.9996** | **1** |
| Filipino | 0.6789 | 0.3458 | 0.2631 | 0.9033 | 0.1217 | **0.9966** | **0.9996** | 0.9557 | 0.9311 | **1** | **1** |
| Hawaiian or other Pacific Islander | 0.6398 | 0.0252 | 0.5199 | **0.0153** | **0.0088** | 0.984 | **0.9985** | 0.8857 | 0.1392 | **0.9999** | **1** |
| Japanese | 0.6111 | 0.0656 | 0.0289 | 0.0271 | **0** | 0.7515 | 0.9154 | 0.5267 | 0.0866 | **0.9988** | **1** |
| Korean | 0.163 | **0.0031** | 0.0012 | **0.0015** | **0** | 0.5972 | 0.9652 | 0.0905 | 0.0041 | **0.9987** | **1** |
| Chinese | 0.5938 | 0.1156 | 0.6283 | 0.0371 | **0.0026** | **0.9944** | **0.9999** | 0.9655 | 0.1291 | **1** | **1** |
| Other Southeast Asian | 0.2015 | 0.0006 | 0.0039 | 0.0443 | **0.0094** | 0.9638 | **0.9993** | 0.7088 | 0.2322 | **1** | **1** |
| Vietnamese | 0.2942 | 0.0431 | 0.3384 | 0.5589 | 0.0508 | **0.9989** | **1** | 0.9442 | 0.8605 | **1** | **1** |
| **Asian and Pacific Islander*** | 0.3868 | **0.0034** | 0.0131 | **0.0173** | **0** | 0.9638 | **0.9996** | 0.2743 | 0.0238 | **0.9999** | **1** |
| European Caucasian | 0.4802 | **0.0111** | 0.3952 | 0.1103 | **0.0109** | **0.9889** | **1** | 0.8189 | 0.1141 | **1** | **1** |
| MidEast/No. Coast of Africa | 0.2228 | **0.0052** | 0.0212 | 0.0649 | **0.0169** | 0.9608 | **0.9995** | 0.3043 | 0.1853 | **1** | **1** |
| **Caucasian*** | 0.6186 | **0.0032** | 0.3025 | 0.0526 | **0.0014** | **0.9941** | **1** | 0.8973 | 0.5137 | **1** | **1** |
| Mexican or Chicano | 0.3063 | **0.0262** | 0.0029 | **0.0036** | **0.018** | 0.9771 | **0.9996** | 0.2362 | 0.0611 | **1** | **1** |
| South/Cntrl Amer. Hisp. | 0.4339 | **0.0111** | 0.0962 | **0.0003** | 0.0071 | 0.9455 | **0.9996** | 0.7997 | 0.0199 | **1** | **1** |
| Caribbean Hispanic | 0.0509 | **0.0012** | 0.0543 | **0.0106** | 0.0041 | 0.7674 | **0.9999** | 0.1011 | 0.0504 | **1** | **1** |
| **Hispanic*** | 0.2668 | **0.007** | 0.0823 | **0.0007** | 0.0047 | 0.8068 | **0.9983** | 0.3181 | 0.1891 | **0.9998** | **1** |
| Caribbean Indian | 0.0264 | **0.0104** | 0.0663 | **0.0093** | 0.0024 | 0.3867 | **0.9925** | 0.2625 | 0.0804 | **1** | **1** |
| North American Indian | 0.4256 | **0.0033** | 0.1624 | 0.0393 | 0.004 | **0.993** | **0.9999** | 0.8152 | 0.031 | **1** | **1** |
| **Native American Indian*** | 0.4554 | **0.0174** | 0.0947 | **0.0107** | 0.0169 | **0.9907** | **0.9998** | 0.8029 | 0.0896 | **1** | **1** |

Broad race groups, composed of the detailed race groups above it in the table
